# The Atypical Ubiquitin E2 Conjugase UBE2L3 Is an Indirect Caspase-1 Target and Controls IL-1β Secretion by Inflammasomes

**DOI:** 10.1016/j.celrep.2017.01.015

**Published:** 2017-01-31

**Authors:** Matthew J.G. Eldridge, Julia Sanchez-Garrido, Gil Ferreira Hoben, Philippa J. Goddard, Avinash R. Shenoy

**Affiliations:** 1 MRC Centre for Molecular Bacteriology and Infection, Imperial College London, London SW7 2AZ, UK

**Keywords:** inflammasomes, caspase-1, IL-1β, UBE2L3, ubiquitin, proteasome, caspase-4, *Salmonella*, *Listeria*, commensals

## Abstract

Caspase-1 activation by inflammasome signaling scaffolds initiates inflammation and antimicrobial responses. Caspase-1 proteolytically converts newly induced pro-interleukin 1 beta (IL-1β) into its mature form and directs its secretion, triggering pyroptosis and release of non-substrate alarmins such as interleukin 1 alpha (IL-1α) and HMGB1. While some caspase-1 substrates involved in these events are known, the identities and roles of non-proteolytic targets remain unknown. Here, we use unbiased proteomics to show that the UBE2L3 ubiquitin conjugase is an indirect target of caspase-1. Caspase-1, but not caspase-4, controls pyroptosis- and ubiquitin-independent proteasomal degradation of UBE2L3 upon canonical and non-canonical inflammasome activation by sterile danger signals and bacterial infection. Mechanistically, UBE2L3 acts post-translationally to promote K48-ubiquitylation and turnover of pro-IL-1β and dampen mature-IL-1β production. UBE2L3 depletion increases pro-IL-1β levels and mature-IL-1β secretion by inflammasomes. These findings regarding UBE2L3 as a molecular rheostat have implications for IL-1-driven pathology in hereditary fever syndromes and in autoinflammatory conditions associated with *UBE2L3* polymorphisms.

## Highlights

• Caspase-1 inflammasomes induce loss of UBE2L3 in macrophages and dendritic cells
• UBE2L3 loss is proteasome-dependent, ubiquitin- and pyroptosis-independent
• UBE2L3 participates in K48 ubiquitylation and proteasomal turnover of pro-IL-1β
• UBE2L3 modulates levels of pro-IL-1β available for processing by caspase-1

## Graphical Abstract

**Figure:**
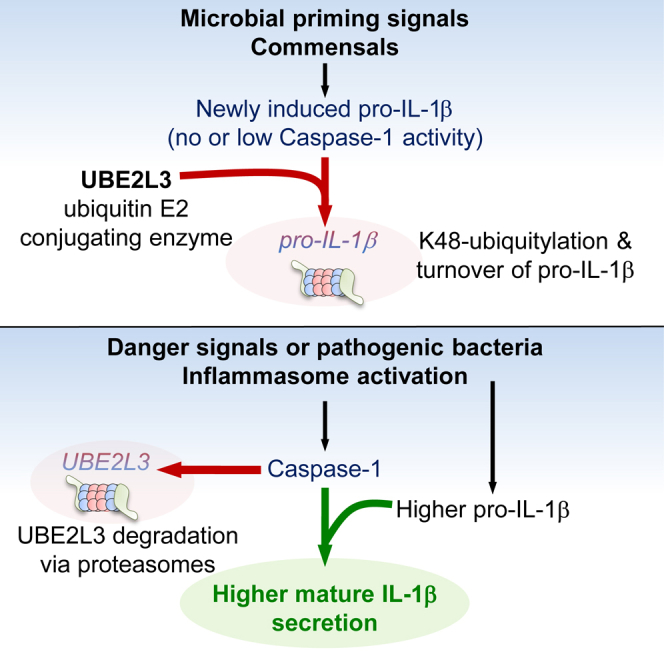

## Teaser

Eldridge et al. show that the UBE2L3 ubiquitin E2 conjugating enzyme is targeted for proteasomal degradation by inflammasomes and is therefore an indirect target of human and mouse caspase-1. They also find that UBE2L3 is a post-translational regulator of pro-IL-1β protein levels and thus regulates mature IL-1β production.

## Introduction

Multi-molecular signaling scaffolds, called inflammasomes, control the cysteine protease caspase-1 (Lamkanfi and Dixit, 2014, von Moltke et al., 2013). Caspase-1 activity is stimulated following the oligomerization of a cytosolic sensor protein into large polymeric inflammasome foci. Sensor proteins include members from the NOD and leucine-rich repeat containing protein (NLRs), AIM2-like receptor (ALRs) families, and Pyrin. NLRs, ALRs, and Pyrin interact with the adaptor protein ASC to recruit and activate caspase-1 within inflammasome foci (Lamkanfi and Dixit, 2014, von Moltke et al., 2013). Complex assembly is triggered by molecular cues from pathogenic and commensal microorganisms (e.g., nucleic acids, bacterial secretion apparatuses, flagellins, and toxins) as well as sterile danger signals and particulates including, ATP, alum adjuvants, and gout-associated uric acid crystals (Eldridge and Shenoy, 2015, von Moltke et al., 2013). In addition, a non-canonical pathway of caspase-1 activation is initiated by caspase-4 (also called caspase-11 in the mouse) or caspase-5, both of which are activated upon binding to cytosolic lipopolysaccharide (LPS) (Hagar and Miao, 2014, Lamkanfi and Dixit, 2014, von Moltke et al., 2013). Actions of caspase-1 include the proteolytic processing of pro-interleukin 1 beta (IL-1β) and pro-interleukin 18 (IL-18) substrates and secretion of their bioactive forms, release of alarmin molecules such as interleukin 1 alpha (IL-1α) and HMGB1, and cell lysis by pyroptosis (Gross et al., 2012, Lamkanfi and Dixit, 2014, von Moltke et al., 2013).

Inflammasome signaling is a tightly controlled two-step process (Lamkanfi and Dixit, 2014). In the first step, called Signal 1, cells are primed by transcriptionally upregulating sensor proteins (e.g., NLRP3 and caspase-4) (Bauernfeind et al., 2009, Rathinam et al., 2012), accessory proteins (e.g., guanylate binding proteins) (Shenoy et al., 2012), and substrates (e.g., pro-IL-1β), or post-translational modification of sensors (e.g., deubiquitylation of NLRP3) (Juliana et al., 2012, Lin et al., 2014). The assembly of inflammasomes is triggered by various second signals (Signal 2). These are exemplified by K^+^ efflux, double-stranded DNA (dsDNA), and bacterial flagellin that activate of NLRP3, AIM2, and NLRC4, respectively (Lamkanfi and Dixit, 2014, Vanaja et al., 2015). Secreted IL-1β, IL-18, IL-1α, and HMGB1 promote inflammatory and antimicrobial responses (Garlanda et al., 2013, Yanai et al., 2012), and pyroptotic death of infected host cells removes microbial replicative niches. However, deregulated inflammasome signaling causes persistent autoinflammation linked to arthritis, gout, diabetes, atherosclerosis, dysbiosis, and cancer (Broderick et al., 2015, Canna et al., 2014, Gagliani et al., 2014). Furthermore, naturally occurring activating mutations in *NLRP3*, *NLRC4*, and *MEFV* (that encodes Pyrin) cause hereditary monogenic fever syndromes driven by excessive IL-1β or IL-18 (Broderick et al., 2015). Remarkably, preventing IL-1β signaling (e.g., with anti-IL-1β or anti-IL-1R therapeutics) relieves clinical symptoms in inflammasome-associated autoinflammatory conditions (Broderick et al., 2015). These findings have highlighted the critical role of IL-1β in chronic inflammation.

Given the physiological importance of inflammasome signaling, it is important to understand the molecular actions of inflammatory caspases more completely. As caspase-1 has robust protease activity, previous studies used peptide-centric proteomics approaches to identify its substrates. Caspase-7, Parkin, NOX2 NADPH oxidase, Rab39A, glycolytic enzymes, and gasdermin-D, among others, have been identified as caspase-1 substrates (Agard et al., 2010, Becker et al., 2009, Erener et al., 2012, Kayagaki et al., 2015, Shao et al., 2007, Shi et al., 2015, Sokolovska et al., 2013, Yu et al., 2014). However, while the cellular actions of caspase-1 substrates have become clearer, the identities and roles of additional targets of caspase-1, such as IL-1α and HMGB1 on which caspase-1 has an indirect effect by controlling their release from cells, remain poorly understood.

In order to find additional targets as well as substrates of caspase-1, we performed an unbiased proteomic analysis, similar to PROTOMAP (Dix et al., 2014), of macrophages following caspase-1 activation. Here, we report UBE2L3 (previously called UBCH7) as a common indirect target of caspase-1 in multiple inflammasome pathways in human and mouse cells. Our results show that UBE2L3 depletion by caspase-1 is required for mature IL-1β production, and in the absence of Signal 2-driven caspase-1 activation, UBE2L3 turns off the highly inflammatory and potentially dangerous pro-IL-1β cytokine.

## Results

### Caspase-1 Activation in Mouse and Human Cells Results in UBE2L3 Depletion Independently of Pyroptosis

**Figure 1:**
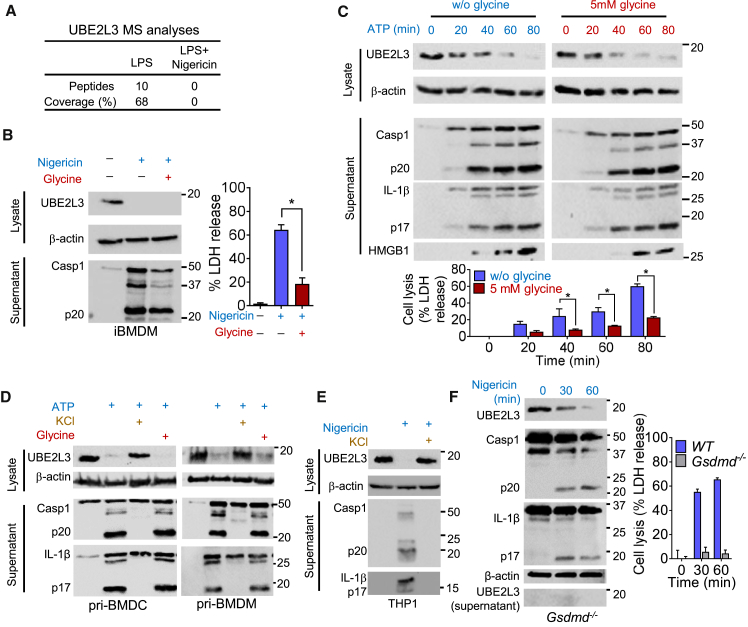
UBE2L3 Is Depleted in Mouse and Human Cells upon Canonical Activation of Caspase-1 by Potassium Efflux iBMDMs (A–C and F), primary BMDC or BMDM (D), or THP1 cells (E) were primed with LPS for 3 hr prior to indicated treatments to activate caspase-1. Immunoblots for UBE2L3 and β-actin in cell lysate and caspase-1, IL-1β, or HMGB1 in culture supernatants are shown (B–E) or in cell lysates for *Gsdmd*^−/−^ cells in (F). (A) Mass spectrometry (MS) results showing the number of peptides and percent coverage of UBE2L3 in cell lysates from LPS-primed iBMDMs left untreated or treated with nigericin for 90 min. (B) UBE2L3 depletes independently of pyroptosis following caspase-1 activation. Immunoblots (left) and cytotoxicity as measured by lactate dehydrogenase (LDH) release (right) following nigericin treatment in the absence or presence of 5 mM glycine. Mean ± SEM from four independent experiments are plotted. ^∗^p < 0.001 by unpaired two-tailed Student’s t test. Data are representative of four experiments. (C) UBE2L3 depletion in iBMDMs temporally correlates with caspase-1 and IL-1β processing and HMGB1 release. ATP treatments were carried out for indicated times without (w/o) or in the presence of 5 mM glycine to prevent pyroptosis. Gels were run and blots developed together; images show similar exposures for both conditions. Plot below shows mean ± SD percent LDH release from two independent experiments. ^∗^BH corrected p < 0.05 by two-way ANOVA. Data are representative of three experiments. (D) UBE2L3 depletes in LPS-primed primary BMDCs and BMDMs treated with ATP. Extracellular KCl inhibits caspase-1 activation, IL-1β processing, and UBE2L3 depletion. Data are representative of two experiments. (E) UBE2L3 depletion and caspase-1 activation in LPS-primed THP1 cells treated with nigericin. Addition of extracellular KCl served as negative control. Data are representative of four experiments. (F) Pyroptosis-independent depletion of UBE2L3 in *Gsdmd*^−/−^ cells. Immunoblots from cell lysates (and supernatants for UBE2L3) of *Gsdmd*^−/−^ cells treated as indicated. Graph on right shows cell lysis (mean ± SD from one of three similar experiments). Data are representative of two experiments.

We carried out comparative mass spectrometric analyses of lysates from LPS-primed immortalized bone marrow-derived macrophages (iBMDMs) left untreated or treated with nigericin to induce K^+^ efflux and caspase-1 activation. Proteomics results were validated based on our identification of known caspase-1 substrates pro-IL-1β and pro-IL-18 and targets such as HMGB1 and IL-1α, which were also depleted after nigericin treatment. Similarly, while ten unique peptides covering 68% UBE2L3 sequence were found in the untreated lysate, none was detected after nigericin treatment (Figures 1A and S1A). In agreement with proteomic analyses, immunoblots consistently revealed that UBE2L3 protein is undetectable in cell lysates upon nigericin-induced caspase-1 activation into its p20-p10 form (Figure 1B). Prolonged caspase-1 activation causes membrane damage and pyroptotic cell death that can be quantified by an assay for lactate dehydrogenase (LDH) in supernatants (Figure 1B). In order to assess whether UBE2L3 was lost passively as a consequence of pyroptosis, we used glycine as an osmoprotectant to reduce pyroptosis as reported previously by multiple groups (Fink and Cookson, 2006, Gross et al., 2012). Glycine plus nigericin treatment reduced pyroptosis by ∼70% in iBMDMs, however, it did not affect the loss of UBE2L3 (Figure 1B). The endogenous danger signal ATP triggers K^+^ efflux through its P2X7 receptor and is a physiological activator of caspase-1. ATP treatment in the absence or presence of glycine resulted in UBE2L3 depletion that temporally correlated with caspase-1 activation, pro-IL-1β processing into mature-IL-1β (p17 form), and HMGB1 release (Figure 1C). Importantly, glycine reduced cell lysis at each time point but had no impact on caspase-1 auto-proteolysis, IL-1β processing, or UBE2L3 depletion (Figure 1C). These results showed that UBE2L3 loss correlates with caspase-1 activation and not pyroptosis. Furthermore, no loss of UBE2L3 was observed in unprimed macrophages treated with nigericin or ATP that do not undergo caspase-1 activation (Figure S1B). UBE2L3 reduction was observed in LPS-primed primary BMDM and bone marrow-derived dendritic cells (BMDC) treated with ATP in the absence or presence of glycine (Figure 1D). Extracellular KCl, which prevents K^+^ efflux and NLRP3-dependent caspase-1 activation, abrogated UBE2L3 depletion (Figure 1D). Thus, little UBE2L3 is present in murine cells following exposure to nigericin or ATP (4%–20% protein detectable at 60 min post treatment; Figures 1A–1D).

UBE2L3 is highly conserved evolutionarily in vertebrates (100% protein identity between human and mouse, 97% with *Xenopus*). We therefore asked whether hUBE2L3 was depleted by caspase-1. Indeed, PMA-differentiated THP-1 macrophage-like cells treated with LPS plus nigericin lost UBE2L3, and extracellular K^+^ prevented both caspase-1 activation and UBE2L3 loss (Figure 1E). Like in murine cells, nigericin-induced UBE2L3 depletion (6%–20% detectable at 60 min post nigericin) in human THP-1 cells temporally correlated with caspase-1 and IL-1β processing (Figure S1C); similar results were obtained with ATP (not shown). Cell death-driven passive protein loss was ruled out as the main cause of reduced UBE2L3 protein by immunoblotting combined preparations of THP1 cell lysate and supernatants that still showed reduced UBE2L3 after caspase-1 activation (Figure S1D). As reported recently (Kayagaki et al., 2015, Shi et al., 2015), stable silencing of gasdermin D (GSDMD) using an optimized miRNA30E plasmid in THP1 effectively blocked pyroptosis (Figure S1E). However, UBE2L3 depletion was unaffected by *GSDMD* silencing (Figure S1E). Similarly, UBE2L3 depletion was intact in LPS plus nigericin treatment of *Gsdmd*^−/−^ cells (Figure 1F), which do not release caspase-1 or IL-1β and are genetically deficient for pyroptotic membrane pore formation and cell death (Kayagaki et al., 2015, Shi et al., 2015). Upon caspase-1 activation in *Gsdmd*^−/−^ cells, UBE2L3 was absent in supernatants but was lost from cell lysates (Figure 1F), which suggested that the loss is likely via an intracellular process. Collectively, these results establish that canonical activation of NLRP3-dependent caspase-1 by K^+^ efflux in human and mouse cells triggers depletion of UBE2L3 that temporally correlates with caspase-1 activation and production of mature IL-1β and is genetically uncoupled from pyroptotic cell death. We therefore decided to investigate UBE2L3 depletion by additional inflammasomes and the underlying mechanisms.

### NLRP3- and AIM2-Inflammasome-Dependent UBE2L3 Depletion Requires Caspase-1

**Figure 2:**
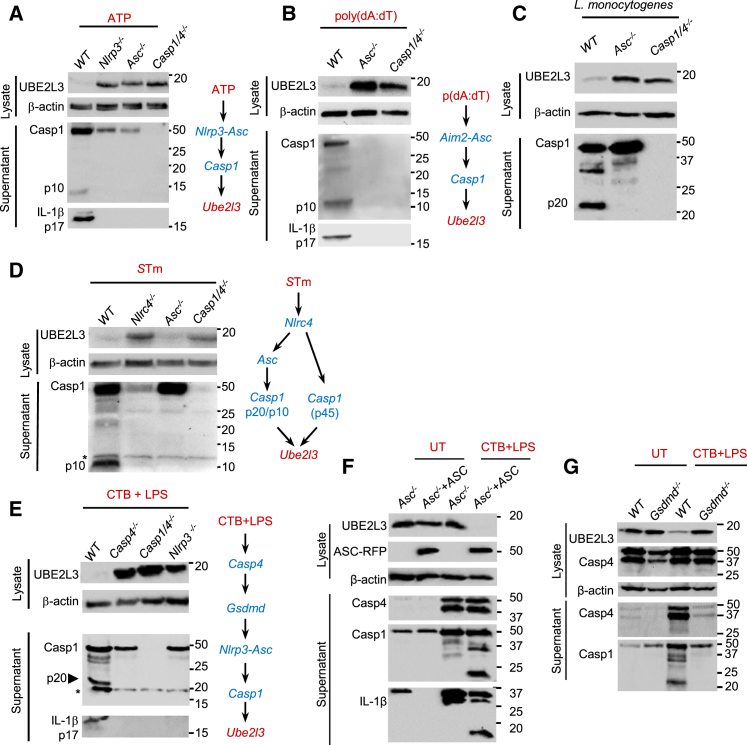
UBE2L3 Depletion by Canonical and Non-canonical Activation of Inflammasomes Is Strictly Caspase-1-Dependent Immunoblots for UBE2L3 and β-actin in cell lysates and caspase-1 (detected with anti-p10 or -p20 antibodies) and IL-1β in culture supernatants from indicated iBMDMs are shown. Cells were primed with LPS for 3 hr (A, C, and D) or PAM3CSK4 for 2 hr (B and E–G) prior to indicated treatments or infections. (A) UBE2L3 does not deplete in *Nlrp3*^−/−^, *Asc*^−/−^, and *Casp1/4*^−/−^ macrophages treated with ATP (left). Schematic (right) shows genes linking the NLRP3 inflammasome to UBE2L3 depletion. Experiments were repeated at least three times. (B) AIM2 activation by poly(dA:dT) transfection fails to deplete UBE2L3 in *Asc*^−/−^ and *Casp1/4*^−/−^ macrophages (left). Schematic (right) shows the involvement of genes linking AIM2 to UBE2L3 depletion. Experiments were repeated at least three times. (C) *L. monocytogenes* infection at a multiplicity of infection (MOI) 40 results in UBE2L3 depletion in a caspase-1-dependent manner. Experiments were repeated at least three times. (D) Infection of *Nlrc4*^−/−^, *Asc*^−/−^, and *Casp1/4*^−/−^cells with *S*Tm (MOI 40) results in *Nlrc4*-dependent and *Asc-*independent UBE2L3 depletion (left). Schematic (right) shows the two possible mechanisms of caspase-1 activation into p45 or p20-p10 forms by NLRC4 during *S*Tm infection. ^∗^Non-specific band. Experiments were repeated at least two times. (E–G) Immunoblots (left) from indicated knockouts (E), *Asc*^−/−^ and *Asc*^−/−^ cells complemented with ASC-RFP (F) or *WT* and *Gsdmd*^−/−^ cells (G) left untreated (UT) or treated with cholera toxin B (CTB, 20 μg/mL) plus LPS (5 μg/mL) for 8 hr. Schematic in (E) shows the non-canonical pathway of LPS-dependent upstream activation of caspase-4 followed by *Nlrp3*- and *Asc*-dependent activation of caspase-1 that targets UBE2L3. ^∗^Non-specific band (E). Experiments were repeated at least two (G) or three (E and F) times.

K^+^ efflux triggered by nigericin, which is independent of the P2X7 receptor of ATP, ruled out a requirement for purinergic signaling in UBE2L3 depletion. Could K^+^ efflux, in the absence of caspase-1 activation, be sufficient for UBE2L3 depletion? We answered this by testing cells deficient in *Nlrp3* or *Asc*; both undergo K^+^ efflux upon ATP and nigericin treatments but fail to activate caspase-1 (Muñoz-Planillo et al., 2013). Treatment of *Nlrp3*^−/−^ and *Asc*^−/−^ iBMDMs with ATP (Figure 2A) or nigericin (Figure S1F) did not deplete UBE2L3; similarly treated *Casp1/4*^−/−^ and *Casp1*^−/−^ iBMDMs retained UBE2L3 protein (Figures 2A, S1F, and S1G). Expression of UBE2L3 in naive or LPS-treated wild-type (WT), *Nlrp3*^−/−^, *Asc*^−/−^, *Casp1*^−/−^, and *Casp1/4*^−/−^ cells is similar (Figures S1G–S1I), however, only wild-type cells undergo loss of UBE2L3 upon caspase-1 activation (Figure 2A). Thus, UBE2L3 depletion is not triggered by K^+^ efflux alone and requires inflammasome-driven caspase-1 activity. Stable silencing of *NLRP3* in THP1 cells blocked nigericin-induced caspase-1 activation as well as loss of UBE2L3 (Figure S2A). Taken together, the canonical NLRP3-ASC-caspase-1 pathway is responsible for UBE2L3 depletion in human and mouse cells. We also observed that exposure to the sterile particulate alum, which activates caspase-1 via cathepsins, NLRP3, and ASC (Hornung et al., 2008), led to UBE2L3 depletion in BMDMs and BMDCs (Figure S2B). Thus, UBE2L3 depletion is a common outcome of canonical NLRP3 inflammasome activation by toxins, danger signals, and sterile particulates.

We then wanted to know whether cytosolic-DNA sensing AIM2 inflammasomes also trigger UBE2L3 depletion. Indeed, AIM2 activation by transfection of poly(dA:dT) resulted in *Asc*- and *Casp1*-dependent loss of UBE2L3 (Figure 2B). AIM2-driven UBE2L3 depletion also occurred in *Gsdmd*^−/−^ cells indicating pyroptosis-independent mechanisms are involved in its loss (Figure S2C). Based on these results, we hypothesized that infection with *Listeria monocytogenes* (*Lm*), a bacterial pathogen that can strongly activate NLRP3 via its listeriolysin toxin, AIM2, by release of genomic DNA, and weakly activate the NLRC4 pathway because of downregulation of flagellin expression (Kim et al., 2010, Warren et al., 2010, Wu et al., 2010), should also cause loss of UBE2L3. In support of this, *Lm* infection resulted in robust caspase-1-dependent UBE2L3 depletion in mouse (Figure 2C) and human (Figure S2D) cells. A listeriolysin O mutant strain of *Lm* (*Lm*Δ*hlyA*), which does not escape from vacuoles, does not stimulate caspase-1 activation or UBE2L3 depletion (Figure S2D). Thus, natural activation of inflammasomes by a bacterial pathogen causes caspase-1-dependent UBE2L3 depletion in human and mouse cells. Furthermore, the *Clostridium botulinum* C3 exotoxin and the anthrax lethal factor that activate the Pyrin and NLRP1 inflammasomes, respectively, also induced marked UBE2L3 depletion (Figures S2E and S2F).

### NLRC4-Dependent UBE2L3 Depletion Is Caspase-1-Dependent and Asc-Independent

Having addressed NLRP3, AIM2 Pyrin, and NLRP1, we postulated that UBE2L3 is also depleted via the NLRC4-caspase-1 pathways activated by several Gram (−ve) bacteria (von Moltke et al., 2013). We tested this by infecting cells with *Salmonella enterica* Typhimurium (*S*Tm) that selectively activates NLRC4 when grown under conditions that induce high expression of the *Salmonella* Pathogenicity Island-1 (SPI-1) type 3 secretion system (T3SS) (von Moltke et al., 2013). While macrophages infected with *S*Tm lost UBE2L3, *Casp1/4*^−/−^ cells retained UBE2L3 protein (Figure S2G); the *S*TmΔ*prgH* mutant lacking the SPI-1 T3SS did not stimulate caspase-1 or deplete UBE2L3 (Figure S2G). Importantly, like NLRP3 and AIM2, UBE2L3 depletion by NLRC4-activating *S*Tm was observed in *Gsdmd*^−/−^ cells, suggesting once again that UBE2L3 depletion proceeds even in the absence of cell lysis (Figure S2H). *Nlrc4*^−/−^ cells, which fail to activate caspase-1 in response to *S*Tm, retained UBE2L3 protein (Figure 2D). Remarkably, however, UBE2L3 did deplete in *S*Tm-infected *Asc*^−/−^ cells (Figure 2D). This could be because NLRC4 can engage caspase-1 via the ASC adaptor or directly via its N-terminal caspase-activation and recruitment domain (see schematic in Figure 2D). *Asc*^−/−^ iBMDMs infected with *S*Tm do not undergo caspase-1 auto-proteolysis into its p20-p10 form (Figure 2D), but they undergo similar pyroptosis as wild-type (WT) BMDMs due to the presence of the p45 form of proteolytically active caspase-1 (Broz et al., 2010). Therefore, wild-type and *Asc*^−/−^ cells mainly differ in the form of active caspase-1 induced by *S*Tm infection. Taken together, NLRC4 inflammasomes stimulate UBE2L3 depletion, and in this scenario, the proteolytically active p45 caspase-1 may be sufficient to induce UBE2L3 depletion.

### Caspase-4 Is Required but Not Sufficient for UBE2L3 Depletion

Having established UBE2L3 depletion by multiple inflammasomes, we asked whether non-canonical activation of caspase-1 by cytosolic LPS also affects UBE2L3 levels. To answer this, we activated caspase-4 by exposing cells to *E. coli* LPS along with cholera toxin B (CTB) that delivers LPS into the cytosol. UBE2L3 depleted in wild-type iBMDMs treated with CTB+LPS, but its levels remained unchanged in *Casp4*^−/−^ and *Casp1/4*^−/−^ cells (Figure 2E). Caspase-4 stimulates downstream activation of caspase-1 by NLRP3 via K^+^ efflux through pores formed by caspase-4-cleaved GSDMD (see schematic in Figure 2E). This raises the possibility that caspase-4 activity could be sufficient to deplete UBE2L3 in the absence of caspase-1. We therefore used *Nlrp3*^−/−^ cells, in which cytosolic LPS activates caspase-4 but not caspase-1, to assess UBE2L3 depletion. UBE2L3 levels depleted to a lesser extent in *Nlrp3*^−/−^ cells treated with CTB+LPS that suggested that caspase-4 activation alone could not efficiently deplete UBE2L3 (Figure 2E). We further assessed this genetically using *Asc*^−/−^ cells that also fail to activate caspase-1 in response to CTB+LPS, and *Asc*^−/−^ cells complemented with ASC-RFP as controls. UBE2L3 depletion was absent in CTB+LPS-treated *Asc*^−/−^ cells (Figure 2F) but was restored in *Asc*^−/−ASC-RFP^ cells (Figure 2F). Importantly, *Gsdmd*^−/−^ cells given CTB+LPS fail to activate caspase-1 and thus retain UBE2L3 protein even though they lose UBE2L3 upon canonical activation of NLRP3, AIM2, or NLRC4 inflammasomes (Figures 1F, S2C, and S2H). These experiments together establish that in the non-canonical pathway, caspase-4 is required for caspase-1 activation but is not sufficient on its own to trigger UBE2L3 depletion in iBMDMs. The specificity of UBE2L3 depletion by caspase-1 was underscored by a lack of its depletion by caspase-8 during apoptosis (Figure S3A).

### UBE2L3 Depletion Requires Cell-Autonomous Activity of Caspase-1 and Proteasomes

**Figure 3:**
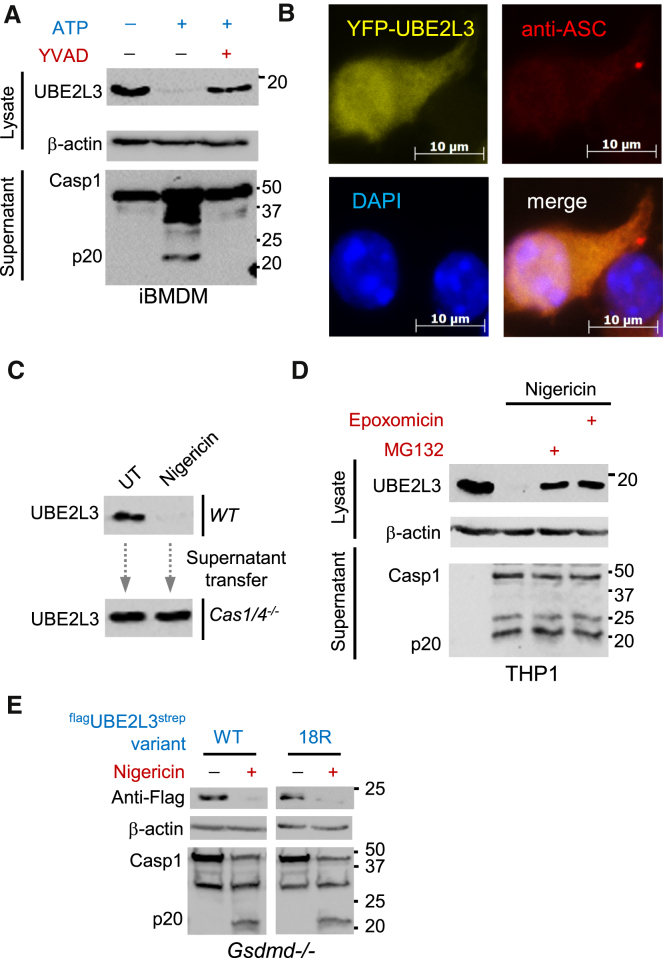
Caspase-1 Activity Is Required for Proteasomal Degradation of UBE2L3 (A) Immunoblots showing UBE2L3 in cell lysates and caspase-1 or IL-1β in culture supernatants from indicated cells. UBE2L3 depletion requires caspase-1 activity in iBMDMs. LPS primed cells were untreated or treated with ATP in the absence or presence of Ac-YVAD-fmk (YVAD). Experiments were repeated at least three times. (B) Immunofluorescence analyses of ASC and UBE2L3 in iBMDMs. YFP-UBE2L3 expressing iBMDMs were treated with LPS plus nigericin and stained with anti-ASC antibody and anti-rabbit-Alexa-647. YFP-UBE2L3 is not recruited to ASC foci in cells. Scale bar, 10 μm. Experiments were repeated at least three times. (C) UBE2L3 depletion requires cell-intrinsic caspase-1 activity. LPS-primed wild-type iBMDMs were treated with nigericin (for 60 min) and supernatants were transferred on to LPS-primed *Casp1/4*^−/−^ cells (for 60 min). Immunoblots for UBE2L3 in cell lysates are shown. Experiments were repeated at least two times. (D) Caspase-1-dependent UBE2L3 depletion requires proteasomal activity. THP1 cells were primed with LPS and left untreated or treated with nigericin as indicated (60 min). DMSO, or proteasomal inhibitors MG132 (20 μM) or epoxomicin (5 μM) were added 5 min after nigericin. Experiments were repeated at least three times. (E) *Gsdmd*^−/−^ cells stably expressing ^flag^UBE2L3^strep^ or a variant with all 18 lysines mutated to arginine (^flag^UBE2L3-18R^strep^) were primed with LPS and left untreated or treated with nigericin. Representative immunoblots performed on cell lysates are shown. A higher exposure of the Anti-Flag blot to detect ^flag^UBE2L3-18R^strep^ is shown due to its poorer expression compared to the wild-type variant. Similar exposures are shown for other blots. Experiments were repeated at least three times.

We first confirmed the requirement of caspase-1 activity and ruled out scaffolding roles of pro-caspase-1 by using the irreversible caspase-1 inhibitor Ac-YVAD-fmk. Notably, inhibition of caspase-1 activity prevented nigericin- or ATP-induced UBE2L3 depletion in human and mouse cells (Figures 3A and S3B). Next, we wanted to know whether UBE2L3 is a proteolytic substrate of caspase-1. This was investigated by two different approaches: first, we tested cleavage of tagged UBE2L3 by caspase-1 in HEK293E cells, and second, we performed in vitro assays using purified recombinant human caspase-1 p20-p10 and GST-UBE2L3 proteins. In silico predictions identified D124 in UBE2L3 as a putative caspase-1 cleavage site, and its mutation to a non-cleavable residue (D124N) was also tested. In both assay systems, however, we were unable to detect cleavage or depletion of UBE2L3 by caspase-1 (Figures S3C–S3F). Further, even though caspase-1 is recruited to ASC-containing specks where it processes pro-IL-1β, we did not detect UBE2L3 recruitment to specks by immunofluorescence analyses (Figure 3B). We therefore concluded that UBE2L3 depletion is an indirect consequence of caspase-1 activation.

Previous reports show that IL-1β or TNF induce late (∼6 hr after treatment) depletion of UBE2D3 (UBCH5C) and UBE2N (UBC13) in mouse embryonic fibroblasts (Shembade et al., 2010). We therefore asked whether UBE2L3 depletion was a result of autocrine/paracrine signaling by caspase-1-dependent cytokines such as IL-1 and IL-18. Importantly, caspase-1-dependent UBE2L3 depletion was observed in *Il1r1*^−/−^ (IL-1α and IL-1β use the same receptor) and *Il18r1*^−/−^ macrophages (Figure S4A). This ruled out IL-1 or IL-18 signaling in UBE2L3 depletion. We then wanted to know whether unknown mediators released after caspase-1 activation could be involved. We hypothesized that *Casp1/4*^−/−^ cells, which cannot produce inflammasome-dependent secreted signals, should still respond to such signals from wild-type cells in *trans*. We therefore tested whether supernatants from inflammasome-activated wild-type cells could trigger UBE2L3 depletion in *Casp1/4*^−/−^ cells. Notably, culture supernatants of LPS plus nigericin-treated wild-type iBMDMs failed to deplete UBE2L3 in *Casp1/4*^−/−^ cells (Figure 3C). This established that *Casp1/4*^−/−^ cells do not deplete UBE2L3 protein upon direct treatment with inflammasome activators (Figure 2) or in response to supernatants from activated wild-type cells (Figure 3C). Taken together, these experiments showed that UBE2L3 depletion requires cell-autonomous caspase-1 activity.

As UBE2L3 is a cytosolic protein involved in protein ubiquitylation, we hypothesized that the autophagy or proteasomes could be involved in its degradation. To address this, we silenced *Atg7* to block autophagy and used MG132 or epoxomicin to inhibit distinct protease activities of the proteasome. *Atg7* silencing did not prevent loss of UBE2L3, ruling out a role for autophagy (Figure S4B). Interestingly, proteasomal inhibitors protected UBE2L3 from depletion in THP1 cells (Figure 3D), iBMDMs, and pri-BMDCs (Figure S4B). This suggested that caspase-1 activity induces proteasomal degradation of UBE2L3. Interestingly, in naive or LPS-treated macrophages, UBE2L3 is a stable protein (half-life >8 hr, Figure S4D), however, caspase-1 activation dramatically reduces its stability and most of the protein is lost by 1 hr (Figures 1C and 1F).

While proteasomal degradation of protein is best characterized for proteins that undergo polyubiquitylation, ubiquitin-independent proteasomal degradation has also been described for several proteins (Ben-Nissan and Sharon, 2014, Inobe and Matouschek, 2014). UBE2L3 has 18 lysine residues, 14 of which were previously identified as ubiquitylated based on di-Gly modifications detected in proteomic analyses of non-phagocytic cells and cancer cell lines (Hornbeck et al., 2015). Importantly, mutation of all 18 lysine residues to non-ubiquitylatable arginine does not affect UBE2L3 folding or catalytic activity in vitro (Hospenthal et al., 2013). We therefore stably expressed wild-type or a non-ubiquitylatable 18K → R variant (UBE2L3^18R^) in *Gsdmd*^−/−^ iBMDMs and assessed their depletion by caspase-1. Remarkably, both wild-type and UBE2L3^18R^ proteins were degraded similarly upon caspase-1 activation (Figure 3E). Interestingly, UBE2L3^18R^ expression was lower in cells and its levels increased upon inhibition of the proteasome (Figure S4E). This further suggested that UBE2L3 is turned over by ubiquitin-independent proteasomal processes during homeostasis. In summary, caspase-1 induces cell-intrinsic, ubiquitin-independent, proteasome-dependent degradation of UBE2L3.

### UBE2L3 Promotes Proteasomal Turnover of Priming-Induced Pro-IL-1β

**Figure 4:**
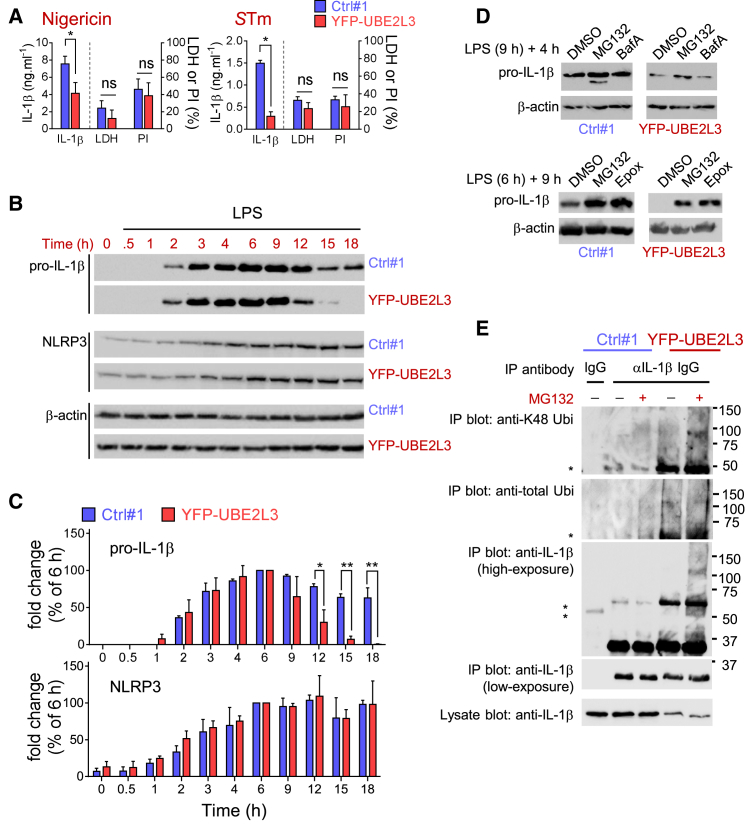
Sustained UBE2L3 Expression Increases Pro-IL-1β Ubiquitylation and Turnover (A) ELISA quantification of IL-1β (left y axis), cell death as measured by release of LDH and uptake of propidium iodide (PI; both on right y axis) in indicated THP1 cells primed with LPS and then treated with nigericin or infected with *S*Tm (MOI 40). Mean ± SEM from three independent experiments are shown. ^∗^BH corrected p < 0.05 by two-way ANOVA; ns, not significant. (B) YFP (Ctrl#1) or YFP-UBE2L3 expressing iBMDMs were treated with LPS (250 ng/mL) for indicated times and UBE2L3, NLRP3, and β-actin were immunoblotted in cell lysates. Data are representative of at least three independent experiments. (C) Quantification of pro-IL-1β relative to β-actin from experiments described in (B). Mean pro-IL-1β/β-actin ratio are plotted as percent of that at 6 hr. Mean ± SEM from three independent experiments are shown. ^∗^BH corrected p < 0.05; ^∗∗^BH corrected p < 0.01 by two-way ANOVA. (D) Indicated iBMDMs were treated with LPS (250 ng/mL) for a total of 13 hr (top) or 15 hr (bottom) in the presence of solvent (DMSO), MG132 (10 μM), bafilomycin A (BafA; 20 nM), or epoxomicin (Epox; 5 μM) as indicated to accumulate pro-IL-1β over a short (4 hr) or long (9 hr) period with inhibitors. Lysates were immunoblotted for pro-IL-1β and β-actin. Data are representative of at least four independent experiments. Images show parts of the same immunoblot with samples from both cell lines; irrelevant intervening lanes were removed. (E) Indicated iBMDMs were primed with LPS (250 ng/mL) for 5 hr and left untreated or treated with MG132 (25 μM) for additional 1 hr, followed by immunoprecipitation (IP) using a normal goat IgG as negative control or goat anti-mouse IL-1β IgG. Cell lysates and IP fractions were immunoblotted with antibodies against K48 polyubiquitin (Ubi), total Ubi, or IL-1β. ^∗^IgG heavy chain. Data are representative of three to four independent experiments.

We hypothesized that UBE2L3 could act as a negative regulator of caspase-1-dependent processes and is therefore targeted for rapid disposal by caspase-1. We asked whether sustained expression of UBE2L3 in the presence of caspase-1 activation could affect caspase-1-dependent processes such as IL-1β processing or pyroptosis. To test this, we wanted to prevent and/or reduce the rate of depletion of UBE2L3 by caspase-1. We noticed that YFP-UBE2L3 depleted to a lesser extent (Figure S5A) than flag-tagged UBE2L3 (Figure 3E), which is presumably due to the larger YFP tag. We took advantage of the increased UBE2L3 stability by YFP-tagging to stably express YFP-UBE2L3 in THP1 and wild-type iBMDMs (iWT cells) for further experiments. As compared to control THP1 cells expressing YFP (THP1^Ctrl#1^), THP1^YFP-UBE2L3^ cells showed diminished LPS-induced pro-IL-1β (Figure S5A) and therefore released ∼2- to 6-fold less IL-1β after nigericin treatment or *S*Tm infection (Figure 4A). Importantly, UBE2L3 overexpression did not affect NLRP3, ASC, or caspase-1 expression or caspase-1 activation (Figures S5A and S5B). This explained why UBE2L3 overexpression did not alter pyroptosis-related LDH release and PI uptake induced by nigericin or *S*Tm (Figure 4A). However, despite similar caspase-1 activation, pro-IL-1β protein in cell lysates depleted faster in THP1^YFPUBE2L3^ as compared to THP1^Ctrl#1^ (Figure S5A). Similarly heightened loss of pro-IL-1β was also seen after caspase-1 activation in THP1 cells expressing ^flag^UBE2L3^HA^ (Figure S5C) that ruled out effects of YFP-tagging. UBE2L3 appeared to act post-transcriptionally as LPS-induced *IL1B* and *TNF* transcript levels were similar 3 hr after LPS treatment (Figure S5D).

Therefore, we asked how UBE2L3 reduced pro-IL-1β protein. LPS-primed iWT^Ctrl#1^ and iWT^YFPUBE2L3^ iBMDMs had comparable pro-IL-1β induction up to 6 hr (Figures 4B and 4C). However, >90% pro-IL-1β protein was lost from iWT^YFPUBE2L3^ cells between 9–18 hr, whereas its expression was retained in control iWT^Ctrl#1^ cells until 18 hr (Figures 4B and 4C). Similarly lower levels of pro-IL-1β were found in LPS-treated *iGsdmd*^−/−^ cells expressing ^flag^UBE2L3^strep^, ruling out epitope or cell line-specific effects (Figure S5E). Pro-IL-1β induced by PAM3CSK4 and TNF was also turned over faster in iWT^YFP-UBE2L3^ cells (Figure S5F), which suggested UBE2L3 acts independently of the upstream priming signal. LPS-induced pro-IL-1α was also lost faster in iWT^YFP-UBE2L3^ cells (Figure S5G). Surprisingly, while pro-IL-1β half-life was reduced, the induction or stability of NLRP3 (Figures 4B and 4C) were unaffected in YFP-UBE2L3 expressing cells. Similar levels of LPS-induced *Il1b*, *Tnf*, and *Il6* mRNA in YFP and YFP-UBE2L3 expressing cells at various times after treatment ruled out priming-related transcriptional effects (Figure S5H); secreted TNF and IL-6 were also comparable (Figure S5I). Caspase-1 activation and pyroptosis were also similar in both cell lines (Figures 4A and S5A–S5C). Therefore, we concluded that UBE2L3 specifically affects the steady-state levels of priming-induced pro-IL-1α/β protein but not cellular priming in general or caspase-1 activation.

Previous reports showed that pro-IL-1β can be ubiquitylated and turned over by proteasomes (Ainscough et al., 2014) or disposed by autophagosomes (Harris et al., 2011). We found that inhibition of proteasomes with MG132 or epoxomycin, but not lysosomal proteases with bafilomycin-A, reduced pro-IL-1β clearance over time in iWT^YFP-UBE2L3^ cells (Figure 4D). Therefore, we asked whether UBE2L3 could be involved in degradative K48-linked polyubiquitylation of IL-1β. To test this, we immunoprecipitated pro-IL-1β at 6 hr after LPS treatment and found that in YFP-UBE2L3 expressing cells, it was associated with significantly higher total and K48 poly-ubiquitin (Figure 4E); K63 polyubiquitin was undetectable (not shown). Thus, sustained UBE2L3 expression promotes K48 ubiquitylation of priming-induced pro-IL-1β and enhances its proteasomal turnover.

### UBE2L3 Downregulates Pro-IL-1β Protein in Response to Commensals and Bacterial Mutants that Cannot Activate Caspase-1

**Figure 5:**
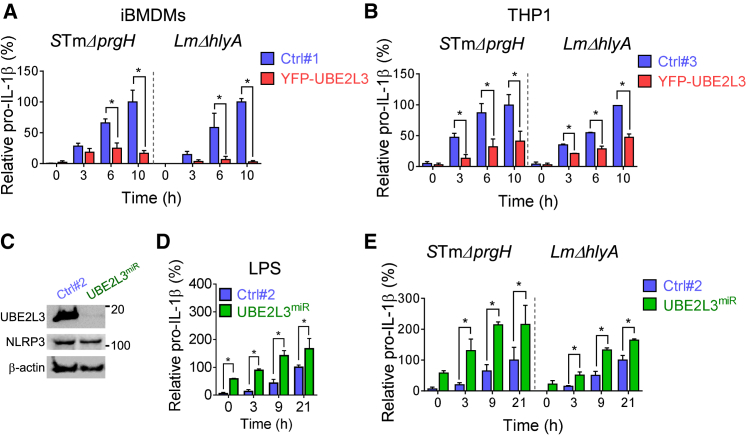
UBE2L3 Controls Pro-IL-1β Levels during Priming and Infection with Bacterial Mutants that Only Provide the Priming Signal (A, B, D, and E) Quantification of relative pro-IL-1β (ratio of pro-IL-1β/β-actin) from western blotting, normalized to levels in control cells at 10 hr (A and B) or 21 hr (D and E) that were taken as 100%. Mean ± SEM from three (A) or four (B) independent experiments or mean ± SD from two experiments (D and E) are plotted. ^∗^BH corrected p < 0.05 by two-way ANOVA for indicated comparisons. (A) Murine iBMDMs expressing YFP (Ctlr#1) or YFP-UBE2L3 were infected with *S*TmΔ*prgH* or *Lm*Δ*hlyA* at MOI 5 and relative pro-IL-1β expression quantified by immunoblots at indicated times. Data are representative of three experiments. (B) THP1 cells expressing YFP (Ctlr#3) or YFP-UBE2L3 were infected with *S*TmΔ*prgH* or *Lm*Δ*hlyA* at MOI 5 and lysates prepared at indicated times. Data are representative of four experiments. (C) Immunoblots show UBE2L3, NLRP3, and β-actin in lysates from LPS-primed THP1 cells expressing non-targeting control (Ctrl#2) or *UBE2L3*-specific miRNA30E (miR). (D) Non-targeting (Ctrl#2) or *UBE2L3* miRNA-expressing THP1 cells were treated with LPS for indicated times and relative pro-IL-1β was quantified by western blotting. (E) Non-targeting (Ctrl#2) or *UBE2L3* miRNA expressing THP1 cells were infected with *S*TmΔ*prgH* or *Lm*Δ*hlyA* at MOI 5 and relative pro-IL-1β expression quantified by immunoblots at indicated times.

As UBE2L3 affected pro-IL-1β protein in response to LPS, PAM3CSK4, and TNF, we asked whether it altered pro-IL-1β produced in response to natural bacterial infection. To avoid pro-IL-1β processing and secretion by caspase-1, we used *S*Tm and *Lm* mutants that do not provide caspase-1 activating Signal 2 and used these bacteria to infect unprimed naive macrophages. As with LPS priming (Figures 4B and 4C), UBE2L3 expression markedly reduced (50%–95% lower) pro-IL-1β accumulation in mouse and human macrophages infected with *S*TmΔ*prgH* or *Lm*Δ*hlyA* (Figures 5A, 5B, and S6A–S6D). This suggested that in the absence of caspase-1 activity, when pro-IL-1β is not being converted into its mature form, UBE2L3 enhances pro-IL-1β turnover and thus switches-off a potentially dangerous pro-inflammatory signal when it might not be required. These experiments also show that human and mouse UBE2L3 function similarly and act in response to both Gram (−ve) and Gram (+ve) bacteria.

To address the role of UBE2L3 in modulating mature IL-1β production, we stably silenced its expression to mimic conditions after caspase-1 activation. Robust *UBE2L3* silencing was achieved (Figure 5C), however, THP1^UBE2L3miR^ grew slowly presumably because UBE2L3 is a cell-cycle-related essential gene based on two recent forward genetic screens (Blomen et al., 2015, Wang et al., 2015). Stable silencing of *UBE2L3* in iBMDMs was not successful due to loss of pools of miRNA expressing cells (data not shown). Interestingly, *UBE2L3* silencing led to ∼6-fold higher levels of LPS-induced pro-IL-1β protein as compared to control cells (THP1^Ctrl#2^, Figures 5D and S6E); LPS-induced NLRP3 expression remained similar (Figure 5C). However, LPS-induced *TNF* mRNA and secreted TNF and IL-6 cytokines remained comparable despite *UBE2L3* silencing (Figures S6F and S6G), which ruled out broad effects on nuclear factor κB (NF-κB)-dependent cytokine production. Pro-IL-1β protein levels upon *UBE2L3* knockdown were significantly higher even though LPS-induced *IL1B* transcriptional induction was slightly reduced over time (Figure S6F). Furthermore, *UBE2L3* knockdown also increased pro-IL-1β accumulation in response to PMA during differentiation of THP-1 cells (Figures 5D and S6E). Higher pro-IL-1β protein was also observed in THP1^UBE2L3miR^ cells infected with *S*TmΔ*prgH* or *Lm*Δ*hlyA* (Figures 5E, S6H, and S6I). Thus, *UBE2L3* silencing has the opposite effect on pro-IL-1β levels as compared to its overexpression (Figures 5A, 5B, 5D, and 5E). Therefore, UBE2L3 is a key post-translational regulator of pro-IL-1β production in both human and mouse cells. Taken together, these findings suggested that caspase-1 acts to swiftly exhaust the cellular pool of UBE2L3 to amplify mature IL-1β production.

### UBE2L3 Depletion Enhances IL-1β Secretion by Inflammasomes

**Figure 6:**
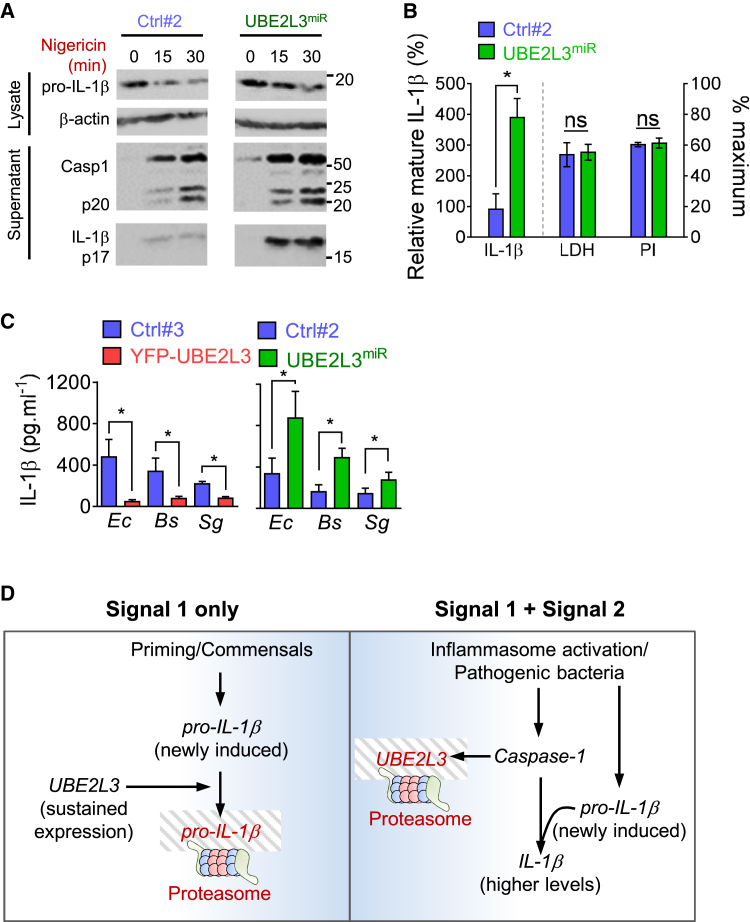
UBE2L3 Depletion Increases Mature IL-1β Production by Inflammasomes (A) LPS-primed THP1 cells expressing non-targeting (Ctrl#2) or *UBE2L3*-specific miRNA were treated with nigericin for indicated times. Immunoblots for pro-IL-1β and β-actin (cell lysates), and caspase-1 and mature IL-1β (culture supernatants) are shown. Data represent three independent experiments. Images show parts of the same immunoblot with samples from both cell lines; irrelevant intervening lanes were removed. (B) Quantification of mature IL-1β released into the supernatant relative to β-actin in cell lysates (left y axis) (mean ± SEM of immunoblots from four independent experiments) and release of LDH and uptake of propidium iodide (PI; right y axis, mean ± SEM from three independent experiments) by indicated LPS-primed THP1 cells treated with nigericin for 60 min. ^∗^BH corrected p < 0.01 by two-tailed unpaired Student’s t test. (C) Indicated THP1 cells were infected with *E. coli* (*Ec*), *B. subtilis* (*Bs*), or *S. gordonii* (*Sg*) for 18 hr at MOI 5 and IL-1β secretion measured by ELISA. Mean ± SEM from three experiments plotted. ^∗^p < 0.05 by paired Student’s t test. (D) Schematic model of UBE2L3 as a rheostat of IL-1β release by inflammasomes. In the presence of only the priming Signal 1 or infection by commensals, UBE2L3 present in cells directs proteasomal turnover of newly induced pro-IL-1β and turns off a potentially dangerous inflammatory signal. In the presence of Signal 2, including toxins or pathogenic bacteria that activate inflammasomes, caspase-1 activation triggers rapid UBE2L3 degradation. Loss of UBE2L3 enhances pro-IL-1β processing and secretion of mature IL-1β by inflammasomes.

As *UBE2L3* silencing did not affect LPS-induced NLRP3 induction, nigericin-induced caspase-1 activation and pyroptosis in THP1^UBE2L3miR^ cells was similar to that in control cells (Figures 6A and 6B). However, *UBE2L3* knockdown led to an ∼6-fold increase in mature IL-1β secretion from THP1^UBE2L3miR^ cells as confirmed by immunoblotting (Figure 6B). Thus, UBE2L3 acts as a negative regulator of mature IL-1β production, and therefore caspase-1 inflammasomes commonly target it for disposal. If this were the case, *UBE2L3* depletion should increase IL-1β production in response to other inflammasomes as well. Indeed, mature IL-1β production by AIM2 activation and non-canonical activation of NLRP3 was enhanced upon *UBE2L3* silencing (Figure S6J).

As the status of host UBE2L3 depends on rapid caspase-1 activation, we also wanted to test its role in response to physiologically relevant commensal bacteria that are poor activators of inflammasomes but provide robust Signal 1 to induce pro-IL-1β expression in naive macrophages. We tested three bacteria that are part of the normal human microbiota, such as *E. coli*, *Bacillus subtilis*, and *Streptococcus gordonii*. IL-1β production at 18 hr post-infection with all three bacteria was markedly reduced in cells overexpressing UBE2L3 and significantly increased in cells in which UBE2L3 expression was silenced (Figure 6C). Taken together, during priming or natural infection with Gram (+ve) or Gram (−ve) pathogenic or commensal bacteria, the UBE2L3 status of host cells sets the upper limit on the amount of pro-IL-1β substrate available for caspase-1 processing and thus controls mature IL-1β production.

## Discussion

We identified UBE2L3 as an indirect caspase-1 target depleted in a pyroptosis-independent, proteasome-dependent manner. We established that UBE2L3 depletion is a requirement for efficient IL-1β secretion by inflammasomes in human and mouse cells (model in Figure 6D). UBE2L3 depletion by caspase-1 occurs in macrophages and dendritic cells after canonical and non-canonical inflammasome activation by microbial or sterile signals and bacterial infection. In this respect, therefore, like cytokine release and pyroptosis, UBE2L3 depletion emerges as a common outcome of inflammasome signaling in general. Mechanistically, UBE2L3 depletion required cell-intrinsic caspase-1 and proteasome activity. Importantly, cellular UBE2L3 levels inversely affected pro-IL-1β and therefore mature IL-1β production (e.g., during activation of multiple inflammasomes, exposure by commensals, or mutants of *S*Tm or *Lm*). In contrast, pathogenic *S*Tm and *Lm* stimulated robust caspase-1 activity and UBE2L3 depletion. Thus, during weak or absent caspase-1 activation, sustained UBE2L3 expression promoted pro-IL-1β turnover (Figure 6D) that could therefore prevent inadvertent inflammation.

The strict requirement for caspase-1 activity—either in its p45 or p20-p10 form—in targeting UBE2L3 was remarkable. In contrast, IL-1β can also be processed by caspase-8 (Gringhuis et al., 2012), IL-18 by caspase-4 (Knodler et al., 2014), and gasdermin-D by both caspase-1 and caspase-4 (Kayagaki et al., 2015, Shi et al., 2015). Similarly, pyroptotic release of IL-1α and HMGB1 can be triggered by caspase-1 or caspase-4 (Gross et al., 2012, Kayagaki et al., 2011). However, neither caspase-4 nor caspase-8 activity alone was sufficient for UBE2L3 depletion in our experiments. The molecular basis for this specificity should become clear from future studies and most likely involves a specific direct substrate of caspase-1.

UBE2L3 operates in the second step of protein ubiquitylation, which is a three-step process involving E1, E2, and E3 enzymes (Berndsen and Wolberger, 2014). Ubiquitin-charged E1 (E1∼ubiquitin) transfers ubiquitin to an E2 to generate the thiol-linked E2∼ubiquitin, and E3 ligases direct the transfer of ubiquitin to substrate proteins. E2 and E3 proteins determine ubiquitin chain linkage type, and target proteins are selected by E3 ligases (Berndsen and Wolberger, 2014). The human genome encodes ∼40 ubiquitin E2 ubiquitin conjugases and >600 E3 ligases (Clague et al., 2015, Wenzel et al., 2011). UBE2L3 is an atypical E2 because structural motifs preclude lysine reactivity and limit ubiquitin transfer to homologous to E6-AP C terminus (HECT) or RING between RING (RBR) families of E3 ligases (Berndsen and Wolberger, 2014, Wenzel et al., 2011). However, UBE2L3 also forms unproductive complexes with several really interesting new gene (RING; >500 family members) E3 ligases; the cellular consequences of these interactions remain unclear (Berndsen and Wolberger, 2014, Wenzel et al., 2011). UBE2L3 did not broadly affect transcriptional priming of macrophages for inflammasome activation or the induction of TNF and IL-6. Experiments that used *UBE2L3* silencing or overexpression together established its role in maintaining pro-IL-1β protein levels post-translationally. Our results are in agreement with a previous report on pro-IL-1β ubiquitylation and turnover by the proteasome (Ainscough et al., 2014) as UBE2L3 promoted K48 polyubiquitylation of pro-IL-1β. However, the E3 ligase involved remains to be identified. Previously studied E3 ligase partners of UBE2L3, such as HOIL-1, Sharpin, A20, or Parkin do not affect priming-induced pro-IL-1β protein levels in macrophages (Duong et al., 2015, Gurung et al., 2015, Rodgers et al., 2014, Vande Walle et al., 2014, Yu et al., 2014). Therefore, systematic RNAi approaches should help identify the E3 ligases that modulate pro-IL-1β protein level.

Our experiments ruled out UBE2L3 as a substrate of caspase-1, a target for autophagy or ubiquitylation-dependent degradation. Our findings are consistent with several mechanisms that could explain loss of cellular UBE2L3. For example, previous studies have shown that disrupting E2-E3 interactions can lead to the proteasomal degradation of E2 enzymes (Shembade et al., 2010). It is possible that caspase-1 proteolytically cleaves an E3 ligase partner of UBE2L3 that disrupts their interaction and might reduce UBE2L3 stability. Our findings support ubiquitin-independent degradation of UBE2L3. It is plausible that ubiquitylation of UBE2L3 is cell-type-specific due to differential expression of E3 ligases. Further, the homeostatic turnover of non-ubiquitylatable UBE2L3 (UBE2L3^18R^ variant) was also proteasome-dependent. Interestingly, ubiquitin-independent degradation has been reported for several common proteins such as IκBε (Xu et al., 2016), IκBα (Fortmann et al., 2015), p21 (Erales and Coffino, 2014), p53 (Tsvetkov et al., 2010), and myelin basic protein (Belogurov et al., 2015), among others (Ben-Nissan and Sharon, 2014, Inobe and Matouschek, 2014). Local disordered regions can target proteins for degradation by 20S proteasomes independently of their ubiquitylation and this can rely on mechanisms such as alternative proteasome subunits or regulatory particles such as the PA28α/β (Ben-Nissan and Sharon, 2014). Moreover, in multimeric proteins, a ubiquitylated subunit can target the degradation of its non-ubiquitylated partner to remodel the protein complex (Prakash et al., 2009). We speculate that a caspase-1-dependent process may induce local unfolding of UBE2L3 and/or ubiquitylation of an E3 ligase partner that may trigger ubiquitin-independent proteasomal loss of UBE2L3. Future work will focus on the identification of a potential degron(s) in UBE2L3 or its binding partners and their regulation by caspase-1.

Single nucleotide polymorphisms (SNP) in *UBE2L3* are associated with autoinflammatory conditions, including systemic lupus erythematosus, Crohn’s disease, rheumatoid arthritis, and celiac disease, among others, which implicate UBE2L3 in inflammatory signaling (Amundsen et al., 2014, Fransen et al., 2010, Lewis et al., 2015, Wang et al., 2012, Ye et al., 2016, Zhang et al., 2015, Zhernakova et al., 2011). NF-κB regulatory roles of UBE2L3 in TNF signaling have been reported in epithelial cells (Fu et al., 2014). SNP genotype-related (rs140490) increase in UBE2L3 expression causes enhanced linear ubiquitylation and NF-κB activity in monocytes and plasma cells, and similar results were obtained by transient transfection of fibroblasts (Lewis et al., 2015). Our results in macrophages and dendritic cells suggest that, like its HOIL-1 and Sharpin partners (Rodgers et al., 2014), UBE2L3 has cell-type-specific functions. Therefore, further studies on post-translational roles of UBE2L3 in other cell types should provide a clearer picture of its role in autoinflammatory scenarios. UBE2L3 is an essential gene (Blomen et al., 2015, Wang et al., 2015), and embryonic stem cells (ESCs) targeting *Ube2l3* are absent in public repositories; therefore, studies on its roles in in vivo mouse models are challenging. However, in the future, UBE2L3-mediated control of IL-1 could be studied in mice using tamoxifen-inducible tissue-specific deletion of a floxed *Ube2l3* locus.

IL-1β is an endogenous pyrogen and has potent inflammatory properties (Broderick et al., 2015, Garlanda et al., 2013). Therefore, the production of mature bioactive IL-1β has multiple checkpoints: first, transcriptional, as its mRNA is not expressed in naive macrophages, second, via the transcriptional regulation of inducible inflammasome proteins such as NLRP3 and caspase-4, and third, post-translational control of caspase-1 activity. NLR/Pyrin-associated hereditary fever syndromes and inflammasome-associated autoinflammation can be clinically improved by blocking IL-1β signaling (Broderick et al., 2015). Our findings with sterile and microbial signals establish that the cellular UBE2L3 status governs the amount of mature IL-1β released by inflammasomes (Figure 6D). Increasing UBE2L3 stability could therefore be a strategy that could be explored for future therapies. In summary, we have identified a molecular rheostat that specifically regulates one arm of the caspase-1 response, and more broadly, we uncovered previously unappreciated cell-intrinsic roles of caspase-1 targets.

## Experimental Procedures

### Cell Treatments and Immunoblots

iBMDMs were grown in DMEM plus penicillin and streptomycin (PS), 10% heat-inactivated fetal bovine serum (HI-FBS), and 20% L929-spent medium. THP1 cells were maintained in RPMI plus PS, HEPES, sodium pyruvate, and 10% HI-FBS, and differentiated with 100 ng/mL phorbol 12-myristate 13-acetate (PMA) for 3 days. Cells were primed with ultrapure O111:B4 LPS (0.25–1.0 μg/mL; 3 hr) or PAM3CSK4 (1 μg/mL; 2 hr), followed by treatment with ATP (5 mM) or nigericin (20 μM) for 60 min or times as indicated. Cells were transfected for 5 hr with poly(dA:dT) (5 μg/mL) or LPS (5 μg/mL) using Lipofectamine 2000. CTB (20 μg/mL) was used along with 5–10 μg/mL LPS for 5 hr. MG132 (20 μM) was added 5 min after ATP or nigericin, or at times as indicated. Recombinant, cell permeable C3 Exoenzyme was used to activate Pyrin and a mixture of anthrax lethal factor and activated protective antigen to activate NLRP1. *S*Tm ATCC 14028s and Δ*prgH* (from David Holden, CMBI) were grown to induce high SPI-1 expression. Briefly, standing cultures of *S*Tm were grown overnight at 37°C in LB containing 300 mM NaCl, re-inoculated into fresh LB-NaCl (1:60 dilution), and incubated in a shaker until OD_600_ ∼0.9–1.2. Bacteria were washed two times in DMEM before use. High SPI-1 expressing *S*Tm were tested to only activate NLRC4 inflammasomes in murine iBMDMs. *Lm* 10403s and Δ*hlyA* (from Angelika Gründling, CMBI) were grown overnight in BHI medium in a shaker at 37°C and washed three times in DMEM before use. Infections were synchronized by centrifuging bacteria on macrophages at 750 × *g* for 10 min. Gentamycin (100 μg/mL) was added 30 min (*S*Tm) or 60 min (*Lm*) post-infection, and cells were incubated further for 2–4 hr. Treatments for immunoblotting were performed in OptiMEM plus sodium pyruvate, followed by precipitation with 4 vol of acetone for 16–20 hr at −20°C. Cell lysates were prepared in 2× Laemmli loading buffer supplemented with complete protease inhibitor and phosphatase inhibitor tablets (Thermo Fisher Scientific), 1 mM PMSF, 10 μM MG132, 10 μM PR619, 10 mM EDTA, and 5% 2-mercaptoethanol. Proteins were separated by SDS-PAGE using Tris-Tricine (for caspase-1 p10) or Tris-Glycine buffer systems and transferred to PVDF membranes (Bio-Rad Laboratories). Additional details are provided in the Supplemental Experimental Procedures.

### Statistical Analyses

All experiments were repeated at least twice. Immunoblot quantification used images acquired on a Chemidoc MP (Bio-Rad), analyzed using Image Lab software (Bio-Rad Laboratories). For ELISA, qRT-PCR, LDH release, and PI uptake assays, two to three technical replicates were used to estimate mean from one experiment. Means from two or more independent experiments were analyzed by two-tailed Student’s t test or two-way ANOVA and the Benjamini-Hochberg (BH) false-discovery rate was used to account for multiple comparisons (Benjamini and Hochberg, 1995). Means that differ at BH corrected p < 0.05 are marked for comparisons indicated in figures. Data plots and statistics used Prism 6 or 7 (Graph Pad Software).

## Author Contributions

Conceptualization, A.R.S.; Investigation, M.J.G.E., J.S.-G., P.J.G., G.F.H., and A.R.S.; Validation, M.J.G.E., J.S.-G., and P.J.G.; Visualization, M.J.G.E., J.S.-G., and A.R.S.; Writing – Original Draft, A.R.S.; Writing – Review & Editing, M.J.G.E. and A.R.S.; Supervision, A.R.S.; Funding Acquisition, A.R.S.

## Supplemental Information

Document S1. Supplemental Experimental Procedures and Figures S1–S6

Document S2. Article plus Supplemental Information
