# Supplementary material for: The Atypical Ubiquitin E2 Conjugase UBE2L3 Is an Indirect Caspase-1 Target and Controls IL-1β Secretion by Inflammasomes
Source: Cell Rep. 2017 Jan 31;18(5):1285–97. doi: 10.1016/j.celrep.2017.01.015 (PMC5300903; doi:10.1016/j.celrep.2017.01.015)
Supplement: Document S1. Supplemental Experimental Procedures and Figures S1–S6 [file mmc1.pdf]

**Cell Reports, Volume *18***

## Supplemental Information

# The Atypical Ubiquitin E2 Conjugase UBE2L3 Is an Indirect Caspase-1 Target and Controls IL-1β Secretion by Inflammasomes

**Matthew J.G. Eldridge, Julia Sanchez-Garrido, Gil Ferreira Hoben, Philippa J. Goddard, and Avinash R. Shenoy**

**SUPPLEMENTAL INFORMATION**

# The atypical ubiquitin E2 conjugase UBE2L3 is an indirect caspase-1 target and controls IL-1β secretion by inflammasomes

Matthew J. G. Eldridge, Julia Sanchez-Garrido, Gil Ferreira Hoben, Philippa J. Goddard, Avinash R. Shenoy*

* corresponding author

**Supplementary Figures 1-7**

**Supplementary Experimental Procedures**

**Supplementary References**

# Figure S1:

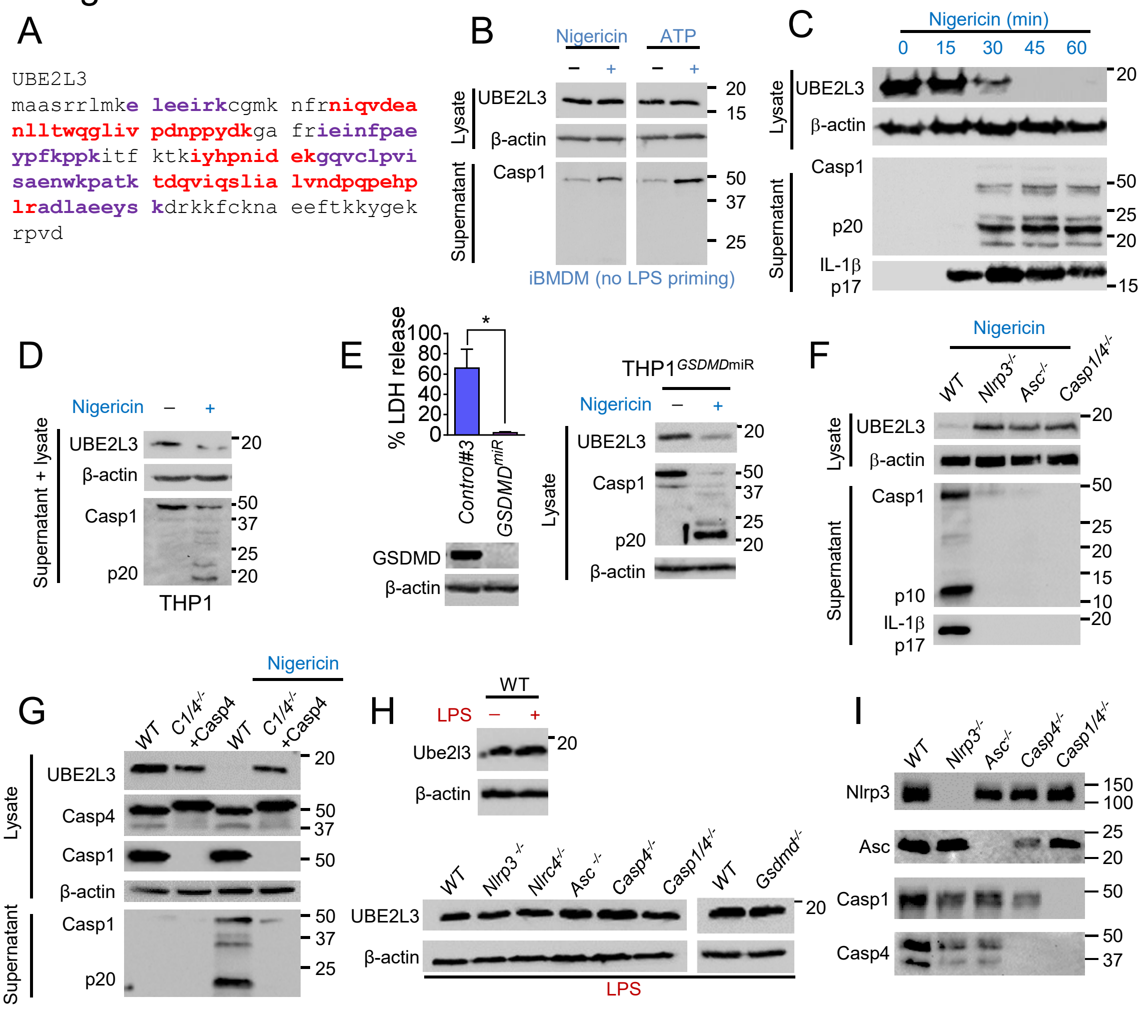


**Figure S1. UBE2L3 peptides identified by proteomics, and its depletion independently of gasdermin-D-driven pyroptosis (related to Figures 1 and 2).**

(*A*) Unique UBE2L3 peptides identified by mass spectrometry from LPS-primed WT iBMDMs following in-gel tryptic digestion are alternatingly coloured purple and red.

(*B*) Unprimed iBMDMs were treated with nigericin or ATP for 1 h followed by immunoblotting as indicated.

(*C*) UBE2L3 depletion in LPS-primed THP1 cells treated with nigericin temporally correlates with IL-1β and caspase-1 processing.

(*D*) Immunoblots from pooled cell lysates and supernatants from LPS-primed THP1 treated as indicated.

(*E*) Stable silencing of *GSDMD* expression with a miR30E (miR) plasmid in THP1 cells blocks pyroptosis as measured by LDH release assays. Mean±S.E.M from three independent experiments plotted. * $P$<0.05 by unpaired, two-tailed Student's *t*-test. Blots show GSDMD knockdown in resting cells, and UBE2L3, caspase-1 and actin from LPS-primed cells left untreated or treated with nigericin.

(*F-G*) UBE2L3 does not deplete in LPS-primed *Nlrp3*$^{-/-}$, *Asc*$^{-/-}$, *Casp1/4*$^{-/-}$ and *Casp1*$^{-/-}$ macrophages treated with nigericin. *Casp1/4*$^{-/-}$ cells were stably transduced with mouse flag-caspase-4 to generate *Casp1*$^{-/-}$ single knockout (*C1/4*$^{-/-}$+Casp4) cells (*G*).

(*H*) Expression of UBE2L3 in WT and indicated iBMDMs untreated or treated with LPS for 3 h.

(*I*) Validation of indicated gene-deficient iBMDMs by immunoblotting.

Experiments in were repeated at least two (*B-D, G-I*) or three (*E-F*) times.

# Figure S2:

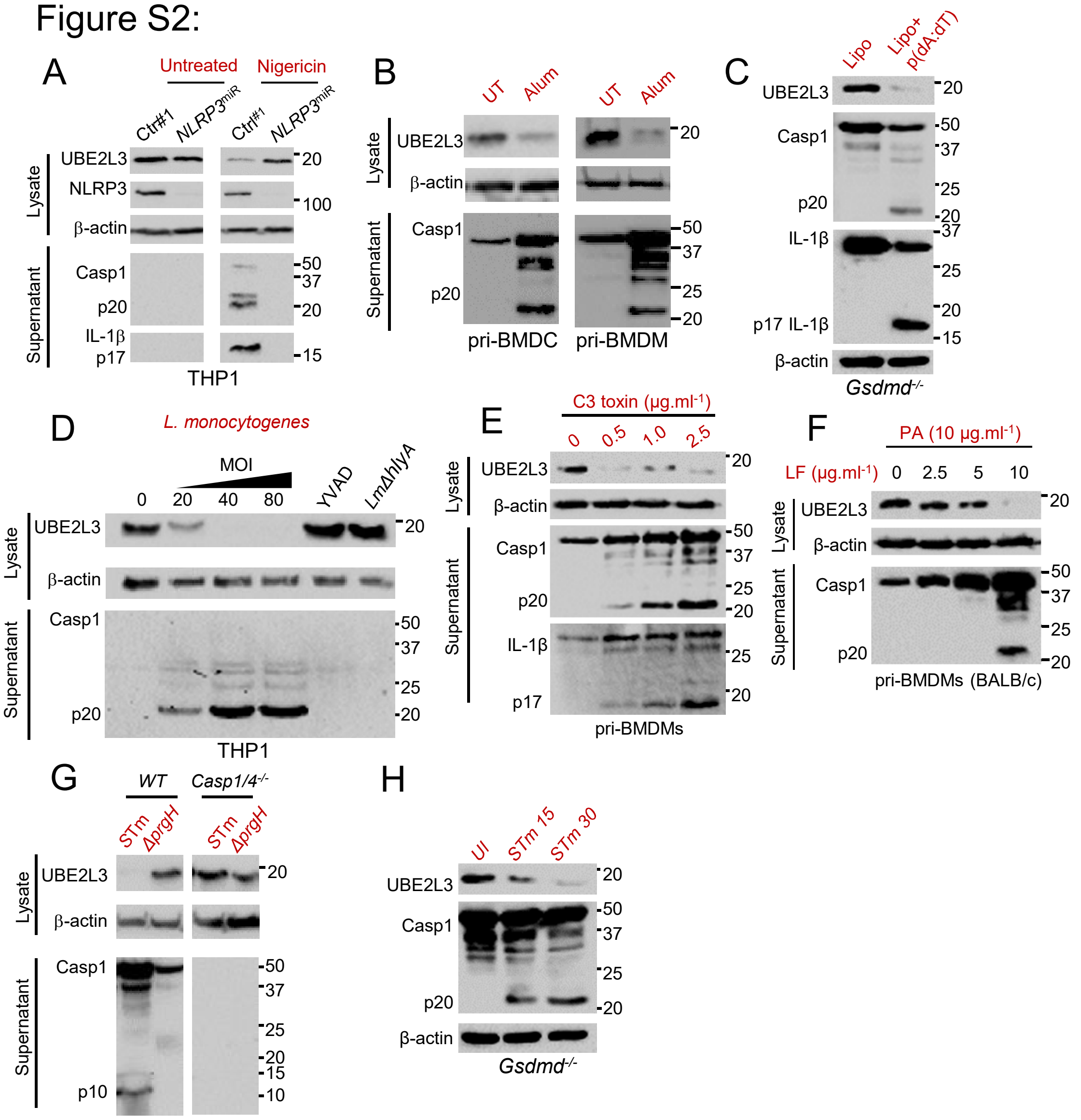


**Figure S2. UBE2L3 depletion during activation of NLRP3, NLRC4, AIM2, Pyrin and NLRP1 inflammasomes (related to Figures 1 and 2).**

(*A*) THP1 stably expressing non-targeting (NT) control or *NLRP3* specific miRNA30E (miR) were primed with LPS and untreated or treated with nigericin, and cell lysates and supernatants immunoblotted. Images are from same immunoblot for indicated antibodies; irrelevant lanes were removed.

(*B*) LPS-primed pri-BMDCs and pri-BMDMs untreated (UT) or treated with alum adjuvant for 5 h show caspase-1 activation and UBE2L3 depletion.

(*C*) PAM3CSK4-primed *Gsdmd*$^{-/-}$ iBMDMs were treated with lipofectamine 2000 (Lipo) alone or along with synthetic p(dA:dT) and lysates were used for indicated immunoblots.

(*D*) *L. monocytogenes* (*Lm*) infection in LPS-primed THP1 cells at indicated multiplicity of infection (MOI) results in UBE2L3 depletion in a caspase-1-activity dependent manner. Ac-YVAD-fmk (YVAD) inhibitor of caspase-1 and *LmΔhlyA* (MOI 80) served as negative controls.

(*E*) LPS-primed primary BMDMs were treated with *Clostridium botulinum* C3 toxin for 4 h at indicated concentrations, and lysates and supernatants used for immunoblotting.

(*F*) LPS-primed primary BALB/c BMDMs were treated with anthrax protective antigen alone or in the presence of indicated concentrations of lethal factor for 6 h. Lysates and supernatants were used for immunoblots.

(*G*) LPS-primed iBMDMs were infected with wild type *S.*Typhimurium (*S*Tm) or a SPI-1 T3SS mutant (*ΔprgH*) that does not activate caspase-1, followed by immunoblotting as indicated. Caspase-1 p10 antibody was used for immunoblots. Images are from same immunoblot for both cell types; irrelevant intervening lanes were removed.

(*H*) LPS-primed *Gsdmd*$^{-/-}$ iBMDMs were left uninfected (UI) or infected with *S*Tm at MOI 15 or 30 for 3 h and indicated immunoblots were carried out on cell lysates.

Experiments in were repeated at least two (*A-C, E, F*) or three (*D, G*) times.

Figure S3:

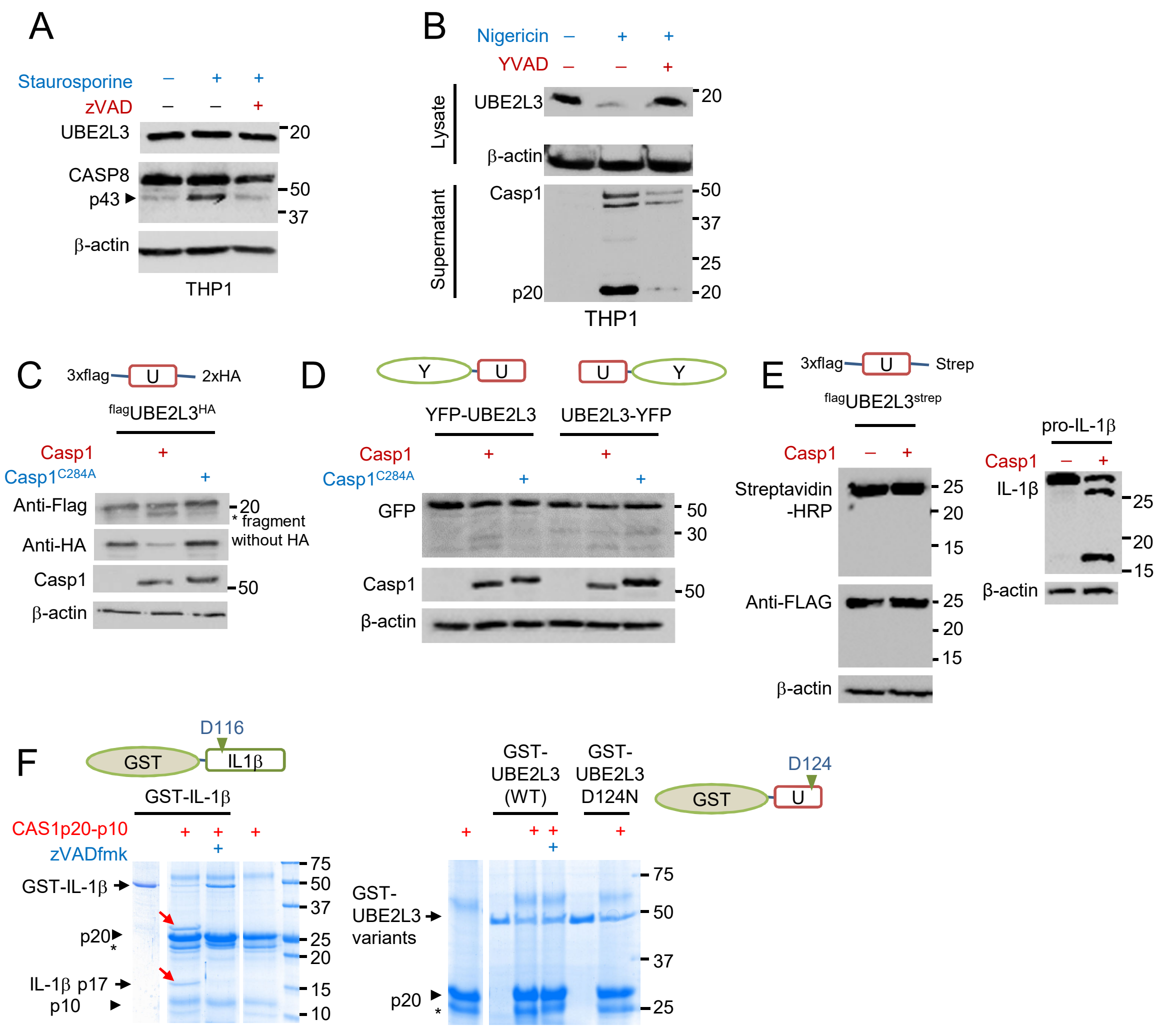


**Figure S3. UBE2L3 is not depleted during apoptosis, and is not a direct substrate of caspase-1 (related to Figures 2 and 3).**

(*A*) THP1 were treated with staurosporine (1 μM) for 3 h in the absence or presence of zVAD (50 μM) to activate apoptosis via caspase-8. Cell lysate UBE2L3 and β-actin are immunoblotted.

(*B*) UBE2L3 depletion requires caspase-1 activity in THP1 cells. LPS primed cells were treated with nigericin in the absence or presence of Ac-YVAD-fmk (YVAD; 50 μM).

(*C-E*) Lack of processing of UBE2L3 by caspase-1. HEK293E cells were transfected caspase-1 or its active-site mutant (C284A) along with $^{flag}$UBE2L3$^{HA}$ (*C*), YFP-UBE2L3 and UBE2L3-YFP (*D*) or $^{flag}$UBE2L3$^{strep}$ (*E*) as indicated. Caspase-1 mediated cleavage of pro-IL-1β as positive control is shown on right in (*E*). Schematics on top show positions of tags, and cleavage by caspase-1 should have resulted in bands of lower apparent $M_w$. Asterisk (*) shows cleaved band as a result of cleavage of the HA tag by caspase-1. Data represent experiments repeated 2-4 times.

(*F*) Recombinant caspase-1 p20 and p10 were used for *in vitro* proteolysis assays using GST-IL-1β (45 kDa) or GST-UBE2L3 (46 kDa) proteins as substrates. Schematics on top show fusion proteins and positions D116 and D124 sites in indicated proteins (not to scale). Coomassie gels of enzyme reactions carried out for 1 h are shown. * indicates spontaneous loss of hexa-histidine tag on p20 protein. zVADfmk was used to inhibit caspase-1. GST-IL-1β is cleaved by caspase-1 to release GST (26 kDa) and p17 IL-1β (indicated by red arrows). Cleavage of GST-UBE2L3 would have led to a smaller fragment but, none was detected. Cleavage at D124 would have produced a ~42 kDa protein. Both assays were carried out at the same time and run on different gels to better separate cleaved bands of varying sizes. Experiments were repeated at least two times.

# Figure S4:

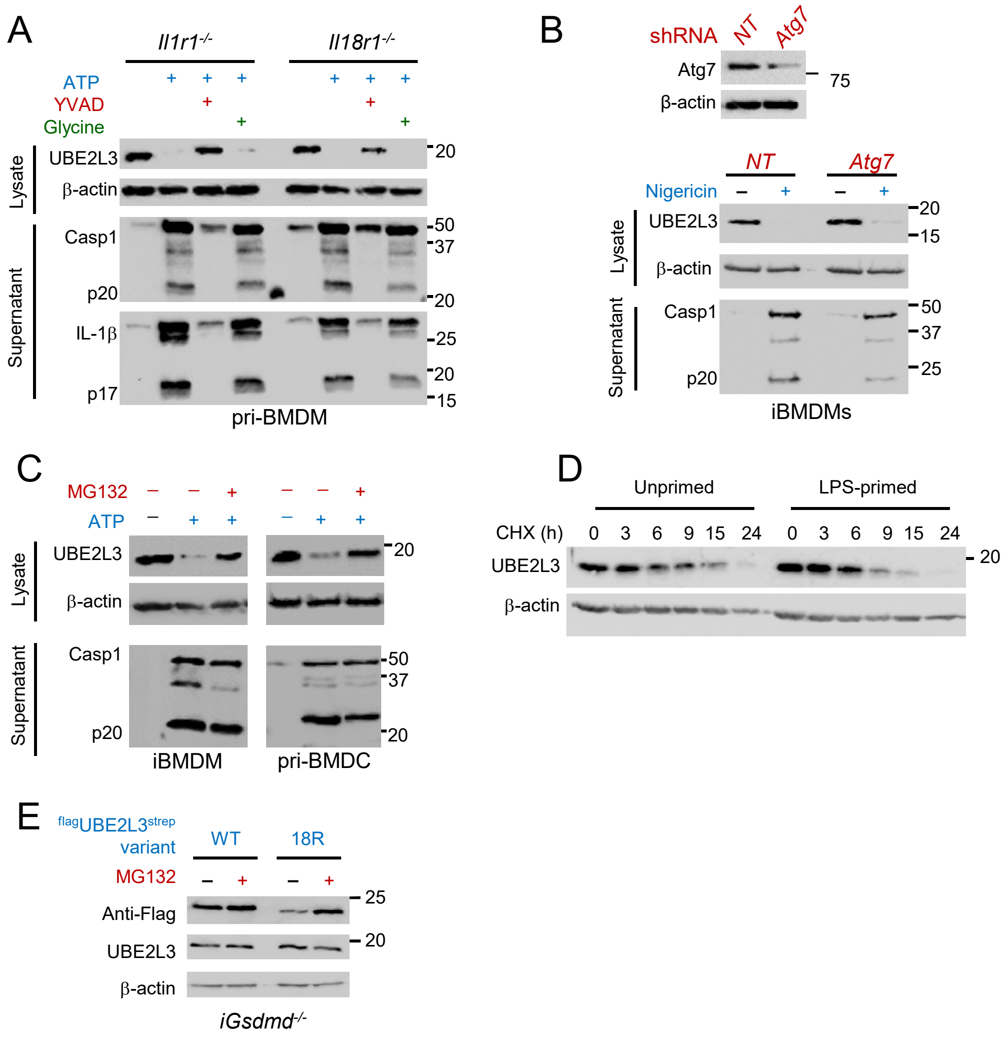


**Figure S4. UBE2L3 is depleted in *Il1r1*$^{-/-}$, *Il18r1*$^{-/-}$ and *Atg7*-silenced cells, and is a stable protein (related to Figure 3).**

(*A*) UBE2L3 depletes in LPS-primed *Il1r1*$^{-/-}$ or *Il18r1*$^{-/-}$ pri-BMDMs treated with ATP in a caspase-1-activity dependent manner. YVAD, Ac-YVAD-fmk.

(*B*) *Atg7* silencing does not prevent loss of UBE2L3. LPS-primed iBMDMs stably expressing non-targeting control (NT) or *Atg7*-specific shRNA were immunoblotted for Atg7 (top) or nigericin for 1 h and lysates and supernatants used for indicated immunoblots.

(*C*) Caspase-1-dependent UBE2L3 depletion requires proteasomal activity. iBMDMs or pri-BMDCs were primed with LPS and left untreated or treated with ATP in the absence or presence of MG132 (20 μM) or DMSO as solvent. MG132 was added 5 min after ATP to prevent inhibition of caspase-1 activation.

(*D*) Unprimed or LPS-primed (3 h) THP1 cells were treated with cycloheximide (CHX, 20 μg.mL$^{-1}$) for indicated times and UBE2L3 and β-actin were immunoblotted in cell lysates.

(*E*) LPS-primed *iGsdmd*$^{-/-}$ cells stably expressing indicated $^{flag}$UBE2L3$^{strep}$ variants were left untreated or treated with MG132 (10 μM) for 6 h and cell lysates prepared for immunoblotting.

Data are representative of experiments repeated two (*A*-*B*) or three (*C*-*E*) times.

Figure S5:

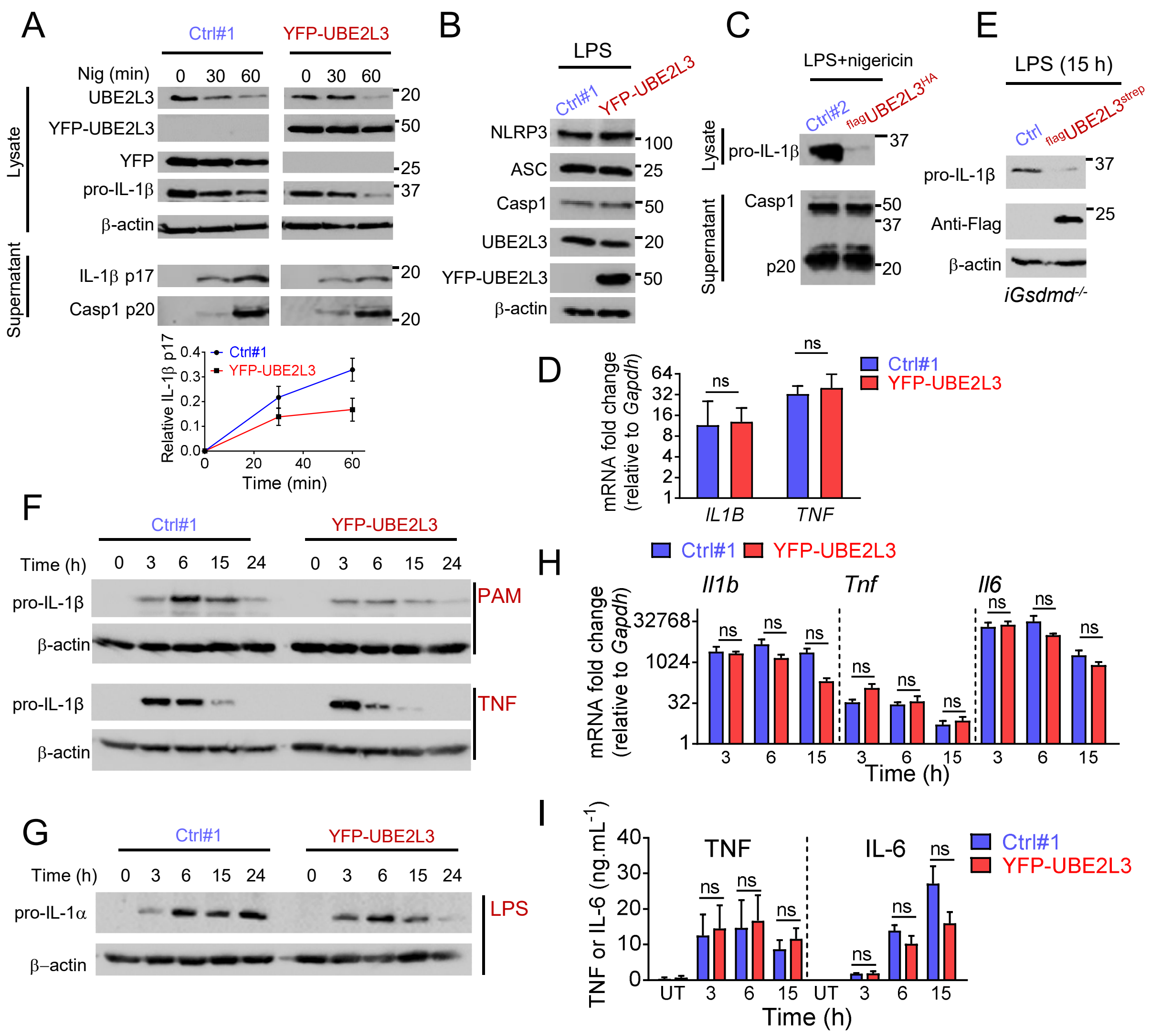

**Figure S5. UBE2L3 determines pro-IL-1β protein levels (related to Figures 4 and 5).**

(*A*) THP1$^{Ctrl\#1}$ and THP1$^{YFPUBE2L3}$ cells were primed with LPS for 3 h and treated with nigericin for indicated times. Indicated proteins were immunoblotted in cell lysates and supernatants. Graph below shows quantification of mature IL-1β from immunoblots of independent experiments (Mean±S.D.). Data are representative of experiments repeated three times. Images for both cell lines are from same immunoblots after removal of intervening lanes.

(*B*) NLRP3, ASC and caspase-1 expression in THP1$^{Ctrl\#1}$ and THP1$^{YFPUBE2L3}$ cells primed with LPS.

(*C*) Immunoblots from THP1$^{Ctrl\#2}$ and THP$^{flag\text{-}UBE2L3\text{-}HA}$ treated with LPS plus nigericin for 60 min.

(*D*) mRNA fold change of *IL1B* and *TNF* in THP1$^{Ctrl\#1}$ and THP1$^{YFP\text{-}UBE2L3}$ expressing cells 3 h after LPS (250 ng.mL$^{-1}$) treatment. Mean±S.E.M. from two independent experiments are shown. ns, not significant.

(*E*) Indicated *Gsdmd*$^{-/-}$ iBMDMs cell lines were treated with LPS for 15 h and cell lysates used for immunoblots.

(*F*) Indicated iBMDMs were treated with PAM3CSK4 (PAM) or TNF for times as shown and pro-IL-1β was detected in cell lysates by immunoblots.

(*G*) Indicated iBMDMs were treated with LPS times as shown and pro-IL-1α was detected in cell lysates by immunoblots.

(*H-I*) *Il1b, Tnf* and *Il6* mRNA fold-change relative to *Gapdh* (*H*) and quantification of secreted TNF and IL-6 by ELISA (*I*) from indicated iWT macrophages left untreated (UT) or treated with LPS (250 ng.mL$^{-1}$) for times as shown. Mean±S.E.M from three independent experiments are plotted. ns, not significant by two-way ANOVA.

Data in *B-I* represent two to three independent experiments.

# Figure S6:

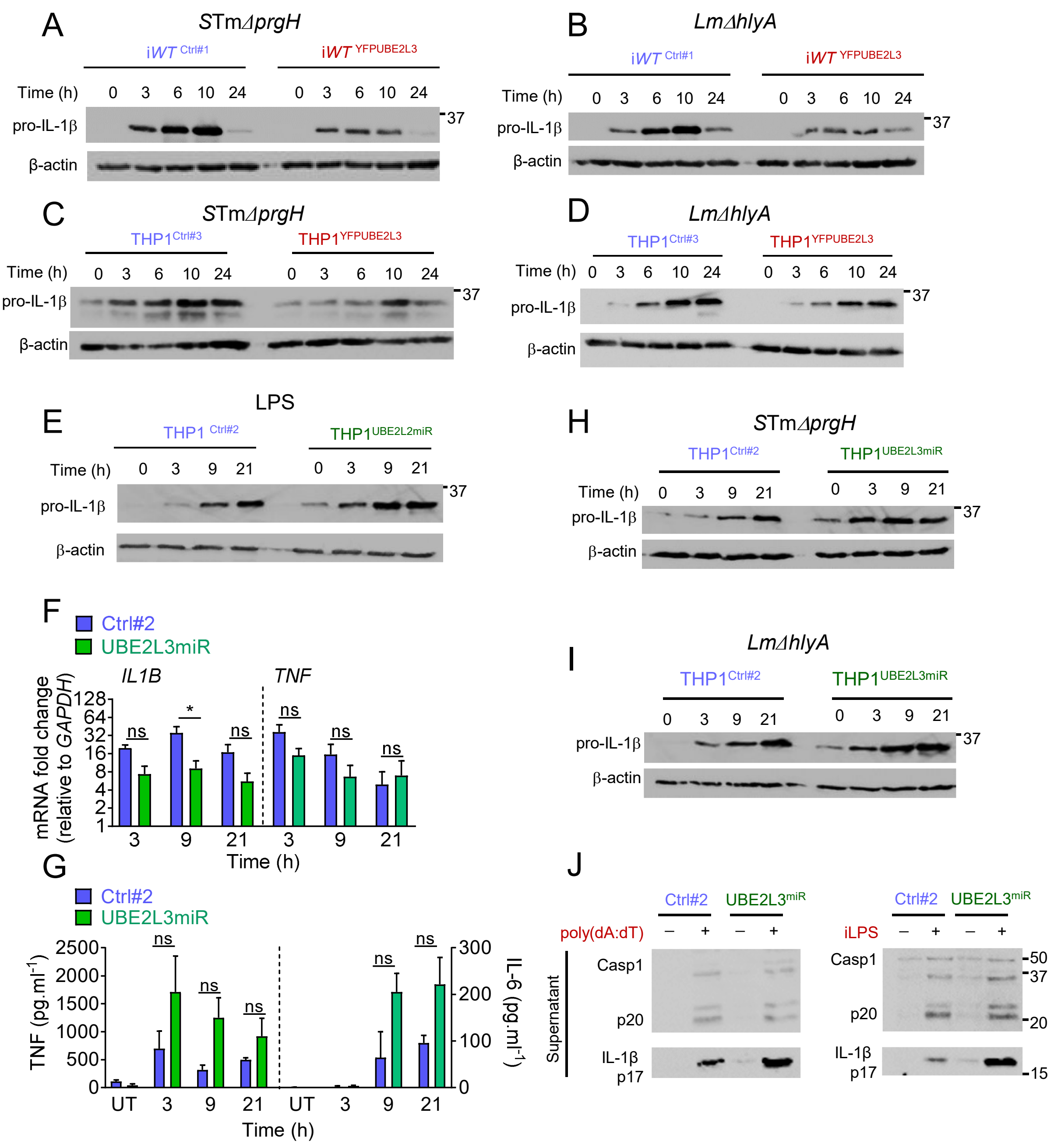


**Figure S6. UBE2L3 determines pro-IL-1β levels when cells are infected with bacteria that only provide the priming signal (Signal 1), and UBE2L3 silencing enhances mature IL-1β production by AIM2 and non-canonical NLRP3 activation (related to Figures 5 & 6)**

(*A-E, H-I*) Representative western blots used for pro-IL-1β quantification in Figure 5 are shown. Macrophages were infected with *STmΔprgH* (*A, C, H*) or *LmΔhlyA* (*B, D, I*) (both at MOI 5) or primed with LPS (*E, F, G*). (*A* and *B)* iWT$^{Ctrl\#1}$ and iWT$^{YFP\text{-}UBE2L3}$, (*C* and *D*) THP1$^{Ctrl\#3}$ and THP1$^{YFP\text{-}UBE2L3}$ and *(E-I)* THP1$^{Ctrl\#2}$ and THP1$^{UBE2L3miR}$ cells. Data are representative of four (*A, B*), three (*C, D, F, G*) or two (*E-H, I*) independent experiments.

(*F-G*) *IL1B* and *TNF* mRNA fold-change relative to *GAPDH* (*F*) and quantification of secreted TNF and IL-6 by ELISA (*G*) from indicated THP1 cells left untreated (UT) or treated with LPS (250 ng.mL$^{-1}$) for times as shown. Mean±S.E.M from three independent experiments are plotted. * BH corrected *P*<0.05 by two-way ANOVA; ns, not significant.

(*J*) Representative western blots used for IL-1β and caspase-1 in supernatants from indicated THP1 cells transfected with poly(dA:dT) or LPS (iLPS) are shown. Cells were primed with PAM3CSK4 for 2 h before transfections. Data represent two independent experiments.

## SUPPLEMENTAL EXPERIMENTAL PROCEDURES

***Reagents***: The following antibodies were used: anti-β-actin-HRP (A3854; Sigma), rabbit anti-Atg7 (D12B11 #8558, Cell Signaling Technologies (CST)), mouse anti-mouse caspase-1 p45 and p20 (Casper-1; Adipogen), mouse anti-human caspase-8 (1C12 #9746, CST), rabbit anti-mouse caspase-1 p45 and p10 (sc-514; SCBT), rabbit anti-human caspase-1 p45 and p20 (D7F10; CST), rat anti-mouse caspase-4/11 (eBioscience; clone 17D9), goat anti-mouse IL-1β (AF401; R&D systems), rabbit anti-HMGB1 (GTX62170; Genetex), mouse anti-human IL-1β (MAB201, R&D systems), mouse anti-NLRP3 (Cryo-2; Adipogen) and rabbit anti-ASC (AL177; Adipogen), rabbit anti-UBE2L3 (GTX104717; GeneTex), mouse anti-UBE2L3 (sc-390032; SCBT; Figure S1D-E, 2F, S2E-F), ubiquitin-HRP (P4D1; SCBT); secondary antibodies from GE Healthcare. ATP, bafilomycin A1, CTB, glycine, MG132, nigericin, pepstatin-A, puromycin, propidium iodide, staurosporine, Ac-YVAD-cmk (all Sigma), epoxomycin (SCBT), zVAD-fmk (FMK001, R&D systems), E64D and PR619 (Calbiochem) were used. Ultrapure *E. coli* O111:B4 LPS, poly(dA:dT) and PAM3CSK4 were from Invivogen, In-gel tryptic digestion kit (#89871), Pierce EDTA-free protease inhibitor tablets (#88666) and Imject Alum Adjuvant (#77161) were from Thermo Fisher Scientific, and recombinant mouse TNF (#14-8321-63) was from eBioscience. C3 Exoenzyme (CT04; Cytoskeleton), and anthrax lethal factor (#169A) and activated protective antigen (#174; both List Laboratories) were used. Immunoblots were developed with Clarity Western (Bio-Rad Laboratories) ECL for cell lysates and ECL Prime (GE Healthcare) for supernatant samples. The following ELISA kits were used: human IL-1β ELISA kit (DY201; R&D Systems), human IL-6 (88-7066; eBioscience), human TNF (88-7346; eBioscience), mouse TNF (88-7324; eBioscience), mouse IL-6 (88-7064; eBioscience). CytoTox96 (#G1780) LDH assay kit was from Promega.

***Mice, iBMDM culture and treatments***: *Nlrp3*$^{-/-}$ mice (Sutterwala et al., 2006) were provided by Richard Flavell, Yale University and Millenium Pharmaceuticals Inc., *Asc*$^{-/-}$ and *Nlrc4*$^{-/-}$ mice (Mariathasan et al., 2004) by Vishwa Dixit, Genentech, and *Casp4*$^{-/-}$ mice (Wang et al., 1998) by Junying Yuan, Harvard University, USA. *Il1r*$^{-/-}$ *and Il18r*$^{-/-}$ were obtained from Jackson labs, and *iGsdmd*$^{-/-}$ cells from Feng Shao, National Institute of Biological Sciences, Beijing, China. *Casp1/4*$^{-/-}$ cells stably expressing flag-tagged mouse caspase-4 were from Teresa Thurston (Thurston et al., 2016). iBMDMs were immortalised using the J2 CRE virus (Blasi et al., 1985) (from Peter Cresswell, Yale University). Briefly, BMDMs from wild-type (C57Bl/6N) or indicated mice were plated in 50% L929-spent medium (L929M) which contains M-CSF and exposed to J2 CRE virus-containing medium on days 5 and 7, followed by continuous culture in reducing L929 spent-medium to a final concentration of 20 percent. iBMDMs were routinely grown in high-glucose DMEM containing penicillin and streptomycin (PS), 10 % heat-inactivated foetal bovine serum (HI-FBS) and 20% L929M, and passaged by trypsinisation. Primary BMDMs were grown similarly in 20% L929M in non-tissue culture treated 10 cm petri-plates and used between 6-15 days. Primary BMDCs were prepared similarly in medium containing GCSF (gift from Gyorgy Fejer, University of Plymouth). Primary BMDMs and BMDCs were detached by removing medium and incubating in ice-cold PBS containing 500 μM EDTA for 20 min. Glycine (5 mM), KCl (50 mM), MG132 (20 μM), Bafilomycin A (20 nM), pepstatin-A (10 $\mu g.mL^{-1}$), E64D (10 $\mu g.mL^{-1}$) and Ac-YVAD-fmk (10 μM for murine cells, 50 μM for THP1) were used as described in figure legends. Cells were plated at a density of 1.5-2 $x10^5$/well in 96 well plates for LDH and PI assays, and 3-4$x10^5$/well in 48 well plates for immunoblots. After treatments, supernatants were removed and used for LDH assay, and PBS containing propidium iodide (5 $\mu g.mL^{-1}$) was added on cells. Cytotoxicity assays used untreated cells (0 %) and 1 % triton-X100 in PBS (100 %) to calculate percent LDH release. Similarly, untreated (0 %) and 0.1 % triton-X100 (100 %) treated cells were used to calculate percent PI uptake. Combined preparations of supernatants and cell lysates were prepared by adding 5x Laemmli buffer containing protease inhibitors, MG132, PR619 and PMSF directly to wells at the end of treatments.

***Infections with commensal bacteria***: *E. coli* ATCC11775 was grown in LB overnight at 37 °C, *Bacillus subtilis* (from Angelika Grundling) was grown overnight at 37 °C in 2X YT broth. *Streptococcus gordonii* (from Andrew Edwards) was grown overnight in Tryptic Soy Broth in a humidified $CO_2$ incubator at 37 °C.

***Cloning and miRNA silencing***: Routine plasmid cloning used sequence and ligation independent cloning (Jeong et al., 2012). pGEX-hUBE2L3 plasmid obtained from Arno Alpi, University of Dundee, was used for PCR (Phusion polymerase, NEB) to generate pMXCMV-YFP-UBE2L3 plasmid. Expression constructs were generated by PCR into the pMXsIP retroviral plasmid. The CMV promoter and enhanced YFP form pEYFP-C1 (Clontech) were cloned into pMXsIP to generate pMXCMV-YFPC1 for increased protein expression. The 3x-flag at N-terminus (from p3Tag1 vector, Agilent) and 2xHA at C-terminus (YPYDVPDYA introduced *via* two PCRs) were added to generate pMXCMV-flag-UBE2L3-HA plasmid. A single Strep-tag II (WSHPQFEK) was introduced by PCR to generate C-terminal strep-tagged proteins. The IRES in pMXCMV was replaced with the PGK promoter from pRetroXTight (Clontech) for increased puromycin expression to generate pMPP-$^{flag}$UBE2L3$^{strep}$ plasmids. The UBE2L3$^{18R}$ variant was custom synthesised by Invitrogen. Mouse caspase-1 (p45 Casp1) used for HEK29E transfections contained the following mutations that abolished self-cleavage: D296N, D308N, D313A and D314A (Broz et al., 2010). DNA fragments cloned by PCR were sequenced prior to use (GATC Biotech). Mutagenesis used single oligonucleotide based linear PCR as described previously (Shenoy and Visweswariah, 2003).

For gene silencing 22 base oligonucleotides (first base mismatch plus 21-mer sense, and 22-mer antisense without mismatches) were cloned in the optimised miRNA30E backbone (Chang et al., 2013; Fellmann et al., 2013). The *Xho*I-*Eco*RI sites in pMXCMV-YFPC1 were used to clone the miRNA30E cassette which also introduced a stop codon in YFP protein. Antisense 22-mer sequences used were: UBE2L3, $^{5'}$TTTCTTTGTAAACTCTTCAGCA$^{3'}$ and $^{5'}$TTTCATCCCACATTTGCGGATT$^{3'}$ adapted from previous reports (Fiesel et al., 2014; Lewis et al., 2015); NLRP3, $^{5'}$AAATTGCGACTCCTGAGTCTCT$^{3'}$ (from TRCN0000431574); GSDMD, $^{5'}$CAGCACCTCAATGAATGTGTA$^{3'}$ (TRCN0000179101); non-targeting control LacZ, $^{5'}$TCACGACGTTGTAATACGACGT$^{3'}$ (TRCN0000072226). Atg7 was silenced using the pLKO.1 shRNA plasmid (TRCN0000092167) and pLXCMV-LacZmiR30E served as non-targeting control. Cells stably transduced with pMXYFP-Ctrl-mi30E which expresses YFP, served as controls; multiple independent stable pools of THP1 and iWT expressing Ctrl-mi30E were generated during the course of studies (Ctrl#1-3; Fig. 3-6), and their responses were similar.

***Retro- and Lenti- viral transduction:*** Virus-like particles were packaged in HEK293E cells using pCMV-MMLV-Gag-Pol (for retroviral plasmids) or pHIV (for lentiviral plasmids) and pseudotyped with pCMV-VSV-G (gifts from Pradeep Uchil and Walther Mothes, Yale University). For packaging, total 1 μg DNA consisting of plasmid-of-interest:Gag-Pol:VSG-G at a ratio of 4:3:2 were transfected with Lipofectamine 2000 for 48 h on cells plated in 1 mL complete medium in 12-well plates. Virus containing supernatants were filtered through 0.45 micron low protein binding filters (Pall Life Sciences) at 48 h post transfection, and 200-400 μL were used to infect target cells in 12-well plates. Puromycin (2 $\mu g.mL^{-1}$ for THP1 or 6 $\mu g.mL^{-1}$ for iBMDMs) was added 48 h after transduction and replenished until stable pools were obtained (1-2 weeks). Cells were sorted on a FACS ARIA III (BD Bioscience) for uniform (>95% +ve) YFP expression.

***Proteomic analysis:*** LPS-primed iBMDMs were treated with nigericin for 90 min and cell lysates were prepared in buffer containing 50 mM Tris-HCl (pH 8.0), 100 mM NaCl, 1 % NP-40, 10 mM DTT and protease inhibitors. Proteins (50 μg) were separated on a 4-20 % gradient gel by SDS-PAGE (Bio-Rad Laboratories) and gels stained with QC Coomassie Colloidal blue staining (Bio-Rad Laboratories). Fourteen 0.5 cm pieces were made of a single lane and untreated and nigericin treated samples were run in parallel as described (http://www.scripps.edu/cravatt/protomap/, (Dix et al., 2014)). Gel bands were destained with ammonium bicarbonate, followed by reduction, alkylation and in-gel tryptic digestion (Thermo Fisher) and analysed on a Synapt G2 (Waters) at the Imperial College Mass Spectrometry Facility. Peptides corresponding to pro-IL-1β (3 peptides, 14.5 % coverage) and HMGB1 (5 peptides, 26 % coverage) were identified in LPS primed cell lysates but, were absent in LPS plus nigericin treated samples. Proteins for which peptides were missing in the nigericin treated samples were analysed further by immunoblotting.

***Recombinant protein production and caspase-1 assays***. UBE2L3 and human IL-1β (aa 110-269) were cloned in pGEX6P (GST tag), and human caspase-1 p20 (aa 120-297) and p10 (aa 317-404) were cloned in pProExHT (N-terminal 6xHis tag). GST-fusion proteins were highly

soluble and purified using standard procedures similar to those described before (Shenoy et al., 2012). Recombinant caspase-1 p20 and p10 were renatured without malonate (Scheer et al., 2005). Briefly, protein expression in *E. coli* RIPL strain was induced with 0.1 mM IPTG for 3 h at 37 °C, cells were sonicated in lysis buffer (50 mM Tris–HCl, pH 8, 150 mM NaCl, and 0.1% Triton X-100, 5 mM DTT). Inclusion bodies were collected by centrifugation at 3000 *xg* for 15 min, washed twice in lysis buffer containing 1% Triton X-100 and denatured in lysis buffer containing 6 M GnHCl overnight. Refolding was carried out by mixing 1 mg each of p20 and p10 rapidly in 100 mL buffer containing 50 mM HEPES, pH 8, 100 mM NaCl, 1 M non-detergent sulfobetaine 201 (NDSB 201) and 10 mM DTT. Precipitates were removed by centrifugation (18,000 *xg*), proteins concentrated by centrifugal concentrators (Millipore), and dialysed overnight against 100 mM HEPES, 10 mM DTT and 10% sucrose. Caspase-1 assay was carried out for 1 h at 37 °C in buffer containing 100 mM HEPES, 0.1 % CHAPS, and 10 mM DTT for 1 h at 37 °C.

***Reverse transcription and qPCR:*** RNA (0.5 – 1 µg) prepared from cells using RNeasy mini kit (Qiagen) or PureLink RNA mini kit (Thermo Fisher Scientific) was used for reverse transcription (RT) using the ProtoScript First Strand cDNA Synthesis Kit (NEB) or TaqMan Reverse Transcription Reagents (Thermo Fisher Scientific) using random hexamer primers. Quantitative PCR were performed using SsoAdvanced Universal SYBR Green Supermix (Bio-Rad Laboratories) on a StepOnePlus Real-Time PCR System (Thermo Fisher Scientific). Fold change in mRNA were calculated by ΔΔCt method normalised to *GAPDH*. Following primer-pairs were used for qPCR:

*hGAPDH*, $^{5'}$TGCCATCAATGACCCCTTC$^{3'}$ , $^{5'}$CTGGAAGATGGTGATGGGATT$^{3'}$;

*hIL1B*, $^{5'}$GACAAAATACCTGTGGCCTTG$^{3'}$, $^{5'}$AGACAAATCGCTTTTCCATCTTC$^{3'}$;

*hTNF,* $^{5'}$ACTTTGGAGTGATCGGCC$^{3'}$, $^{5'}$GCTTGAGGGTTTGCTACAAC$^{3'}$;

*mGapdh*, $^{5'}$AATGGTGAAGGTCGGTGTG$^{3'}$, $^{5'}$GTGGAGTCATACTGGAACAT$^{3'}$;

*mIl1b*, $^{5'}$CTACCTGTGTCTTTCCCGTG$^{3'}$, $^{5'}$TGCAGTTGTCTAATGGGAACG$^{3'}$;

*mTnf*, $^{5'}$AGACCCTCACACTCAGATCA$^{3'}$, $^{5'}$TGTCTTTGAGATCCATGCCG$^{3'}$;

*mIl6,* $^{5'}$CAAAGCCAGAGTCCTTCAGAG$^{3'}$, $^{5'}$GTCCTTAGCCACTCCTTCTG3$^{'}$.

***Immunofluorescence analyses:*** Immunofluorescence staining was carried out as described before (Shenoy et al., 2012). Briefly, cells were plated on coverslips and treated with LPS plus nigericin, followed by fixing in 4 % paraformaldehyde in PBS for 15 min. Coverslips were washed thrice and solubilized with PBS containing 0.3 % triton-X100 for 3 min. Cells were treated with PBS containing 5 mg.mL$^{-1}$ BSA, 10 % donkey serum for 60 min, followed by staining with ASC antibody for 1 h in PBS, 5 mg.mL$^{-1}$ BSA and 0.1 % saponin. Donkey anti-rabbit antibody-Alexa 647 was used as secondary antibody, and nuclei were stained with Hoechst 33342 dye. Coverslips were mounted in ProLong Gold Antifade (Thermo Fisher) and images obtained on a Zeiss inverted microscope using a 100 x oil immersion lens.

***Supplemental References***

Blasi, E., Mathieson, B.J., Varesio, L., Cleveland, J.L., Borchert, P.A., and Rapp, U.R. (1985). Selective immortalization of murine macrophages from fresh bone marrow by a raf/myc recombinant murine retrovirus. Nature *318*, 667-670.

Broz, P., von Moltke, J., Jones, J.W., Vance, R.E., and Monack, D.M. (2010). Differential requirement for Caspase-1 autoproteolysis in pathogen-induced cell death and cytokine processing. Cell Host Microbe *8*, 471-483.

Chang, K., Marran, K., Valentine, A., and Hannon, G.J. (2013). Creating an miR30-based shRNA vector. Cold Spring Harb Protoc *2013*, 631-635.

Dix, M.M., Simon, G.M., and Cravatt, B.F. (2014). Global identification of caspase substrates using PROTOMAP (protein topography and migration analysis platform). Methods Mol Biol *1133*, 61-70.

Fellmann, C., Hoffmann, T., Sridhar, V., Hopfgartner, B., Muhar, M., Roth, M., Lai, D.Y., Barbosa, I.A., Kwon, J.S., Guan, Y., *et al.* (2013). An optimized microRNA backbone for effective single-copy RNAi. Cell Rep *5*, 1704-1713.

Fiesel, F.C., Moussaud-Lamodiere, E.L., Ando, M., and Springer, W. (2014). A specific subset of E2 ubiquitin-conjugating enzymes regulate Parkin activation and mitophagy differently. J Cell Sci *127*, 3488-3504.

Jeong, J.Y., Yim, H.S., Ryu, J.Y., Lee, H.S., Lee, J.H., Seen, D.S., and Kang, S.G. (2012). One-step sequence- and ligation-independent cloning as a rapid and versatile cloning method for functional genomics studies. Appl Environ Microbiol *78*, 5440-5443.

Lewis, M.J., Vyse, S., Shields, A.M., Boeltz, S., Gordon, P.A., Spector, T.D., Lehner, P.J., Walczak, H., and Vyse, T.J. (2015). UBE2L3 polymorphism amplifies NF-kappaB activation and promotes plasma cell development, linking linear ubiquitination to multiple autoimmune diseases. Am J Hum Genet *96*, 221-234.

Mariathasan, S., Newton, K., Monack, D.M., Vucic, D., French, D.M., Lee, W.P., Roose-Girma, M., Erickson, S., and Dixit, V.M. (2004). Differential activation of the inflammasome by caspase-1 adaptors ASC and Ipaf. Nature *430*, 213-218.

Scheer, J.M., Wells, J.A., and Romanowski, M.J. (2005). Malonate-assisted purification of human caspases. Protein Expr Purif *41*, 148-153.

Shenoy, A.R., and Visweswariah, S.S. (2003). Site-directed mutagenesis using a single mutagenic oligonucleotide and DpnI digestion of template DNA. Anal Biochem *319*, 335-336.

Shenoy, A.R., Wellington, D.A., Kumar, P., Kassa, H., Booth, C.J., Cresswell, P., and MacMicking, J.D. (2012). GBP5 promotes NLRP3 inflammasome assembly and immunity in mammals. Science *336*, 481-485.

Sutterwala, F.S., Ogura, Y., Szczepanik, M., Lara-Tejero, M., Lichtenberger, G.S., Grant, E.P., Bertin, J., Coyle, A.J., Galan, J.E., Askenase, P.W., *et al.* (2006). Critical role for NALP3/CIAS1/Cryopyrin in innate and adaptive immunity through its regulation of caspase-1. Immunity *24*, 317-327.

Thurston, T.L., Matthews, S.A., Jennings, E., Alix, E., Shao, F., Shenoy, A.R., Birrell, M.A., and Holden, D.W. (2016). Growth inhibition of cytosolic *Salmonella* by caspase-1 and caspase-11 precedes host cell death. Nat Commun *7*, 13292.

Wang, S., Miura, M., Jung, Y.K., Zhu, H., Li, E., and Yuan, J. (1998). Murine caspase-11, an ICE-interacting protease, is essential for the activation of ICE. Cell *92*, 501-509.
